# Region- and Cell-type–Resolved Multiomic Atlas of the Heart

**DOI:** 10.1016/j.mcpro.2025.100922

**Published:** 2025-02-05

**Authors:** Fan Zhang, Yunzhi Wang, Jiajun Zhu, Jinxi Wang, Qiang Li, Jinwen Feng, Mingwei Liu, Kai Li, Jiliang Tan, Rongkui Luo, Huangtian Yang, Yingyong Hou, Fuchu He, Jun Qin, Chen Ding, Wenjun Yang

**Affiliations:** 1 Department of Pediatric Orthopedics, Xin Hua Hospital Affiliated to Shanghai Jiao Tong University, School of Medicine, Shanghai, China; 2 State Key Laboratory of Genetic Engineering, School of Life Sciences, Human Phenome Institute, Zhongshan Hospital, Fudan University, Shanghai, China; 3 Department of Pathology, Duke University School of Medicine, Durham, North Carolina, USA; 4 Laboratory of Molecular Cardiology, CAS Key Laboratory of Tissue Microenvironment and Tumor, Shanghai Institute of Nutrition and Health, University of Chinese Academy of Sciences (CAS), CAS, Shanghai, China; 5 State Key Laboratory of Proteomics, National Center for Protein Sciences (Beijing), Beijing Institute of Lifeomics, Beijing, China; 6 Departments of Cancer Research Institute, Affiliated Cancer Hospital of Xinjiang Medical University, Xinjiang Key Laboratory of Translational Biomedical Engineering, Urumqi, China

**Keywords:** all-trans retinoic acid, cell-type-resolved proteome, dilated cardiomyopathy, heart, region-resolved proteome

## Abstract

The heart is a vital muscular organ in vertebrate animals, responsible for maintaining blood circulation through rhythmic contraction. Although previous studies have investigated the heart proteome, the full hierarchical molecular network at cell-type– and region-resolved level, illustrating the specialized roles and crosstalk among different cell-types and regions, remains unclear. Here, we presented an atlas of cell-type–resolved proteome for mouse heart and region-resolved proteome for both mouse and human hearts. In-depth proteomic analysis identified 11,794 proteins across four cell-types and 11,995 proteins across six regions of the mouse heart. To further illustrate protein expression patterns in both physiological and pathological conditions, we conducted proteomic analysis on human heart samples from four regions with dilated cardiomyopathy (DCM). We quantified 8201 proteins in DCM tissue and 8316 proteins in adjacent unaffected myocardium tissue across the four human heart regions. Notably, we found that the retinoic acid synthesis pathway was significantly enriched in the DCM-affected left ventricle, and functional experiments demonstrated that all-trans retinoic acid efficiently rescued Ang II–induced myocardial hypertrophy and transverse aorta constriction–induced heart failure. In conclusion, our datasets uncovered the functional features of different cell-types and their synergistic cooperation centered by cell-type–specific transcription factors (TFs) in different regions, while these TF–TG (target gene) axes were significantly altered in DCM. Additionally, all-trans retinoic acid was demonstrated to be an efficient treatment for heart failure. This work presented a panoramic heart proteome map, offering a valuable resource for future cardiovascular research.

## Highlights

• Region- and cell-type-resolved multiomic atlas of physiologic heart is depicted.
• Defined the function division of the four cell-types and six regions of heart.
• Established a hierarchical transduction network between CMs and nonmyocytes.
• Drew a proteome landscape of 12 dilated cardiomyopathy.
• All-trans retinoic acid has a potential therapeutic effect in heart failure.

## Graphical Abstract

**Figure:**
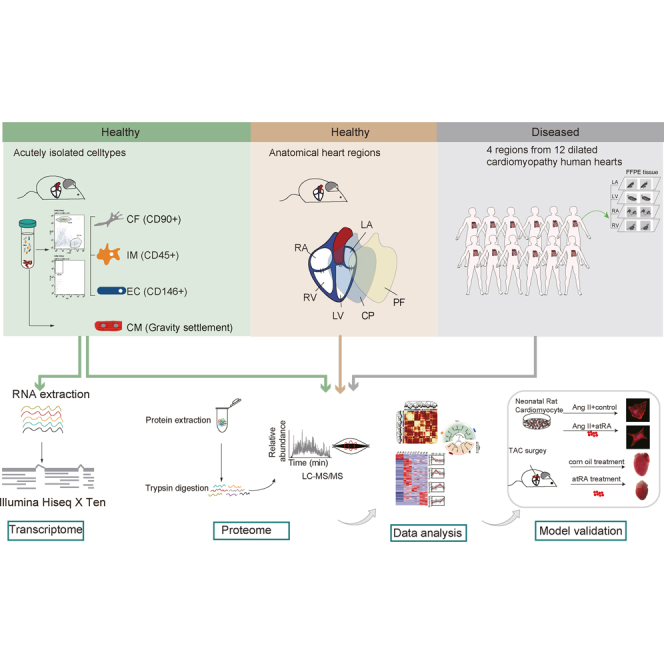

## In Brief

This work presented region- and cell-type-resolved multiomic atlas of physiologic heart. We established a hierarchical transduction network between myocytes and non-myocytes, and defined the function division of the heart. We further performed a proteome in 12 patients of dilated cardiomyopathy (DCM). The data revealed the synthesis pathway of retinoic acid was significantly enriched in DCM area, and further functional experiments demonstrated all-trans retinoic acid can efficiently rescue Ang II induced myocardial hypertrophy and transverse aorta constriction induced heart failure.

The heart is the crucial organ responsible for circulating oxygenated blood and nutrients throughout the body. It is composed of multiple cell-types, including cardiomyocytes (CMs), cardiac fibroblasts (CFs), endothelial cells (ECs), and immune cells (IMs) (1). Cardiomyocytes constitute approximately 30% of the heart’s cell population and 75% of heart’s volume (2). As the heart's parenchymal cells, CMs play a dominant role in functions such as oxidative phosphorylation, electrical impulses, mechanical contraction, and blood supply. However, other cell-types also perform significant regulatory functions. For instance, CFs are crucial for extracellular matrix (ECM) homeostasis, fibrosis formation, and myocardial remodeling (3, 4, 5, 6). ECs, the most abundant cell-type, form the interior blood vessels and valves, secreting cytokines and growth factors that regulate cardiac homeostasis and disease (7, 8). IMs are also abundant in heart tissue, acting as regulators of biological processes and defending against exogenous stimuli (9, 10). Heart function is orchestrated by complex and dynamic crosstalk among different cell-types, which has been shown to vary with the cardiac development, physiological changes, and different types of pathology (11, 12). Different cell-types cooperate together to preform and maintain the heart functions. For example, ECs have been implicated as the first responders to the stimulation which then transduce the signals to the CMs. The ECs secrete heparanase, which in turn triggers the secretion of lipoprotein lipase and vascular endothelial growth factor (VEGF) by CMs. As a feedback, the VEGF have a paracrine influence on the ECs, to facilitate angiogenesis through endothelial migration and proliferation (13, 14). The direct cell–cell interaction between CFs and CMs is crucial to promote and maintain CMs’ function by enhancing the expression of essential cardiomyocyte genes (15). In the anatomic aspect, the heart can be divided into distinct regions such as left atrium (LA), left ventricle (LV), right atrium (RA), right ventricle (RV), cardiac pericardium (CP), and pericardial fat (PF), each with unique cellular compositions and molecular signatures.

Given the heart’s complexity, dysfunction in any cell-type or region can lead to systemic heart failure (16, 17, 18). Dilated cardiomyopathy (DCM) is characterized by ventricular chamber enlargement and impaired blood pumping, primarily affecting the LV, and is a leading cause of heart failure (19). While previous research has revealed significant genetic alterations in DCM, such as changes in RBM20, TTN, LAMA2, and TBX20, the region-specific proteomic patterns in DCM remain inadequately explored (20). Since cardiomyopathy often manifests region-specific pathological phenotypes, it is essential to investigate the regional proteomic profiles and interactions among different cell-types within specific regions to better understand disease progression and prognosis.

Mass spectrometry (MS)-based proteomics is a powerful tool for uncovering spatiotemporal protein expression patterns. In previous studies by our and other groups, the cell-type– and region-resolved organ proteomes have been characterized in the liver, eye, and brain (21, 22, 23). For the heart organ, Quaife-Ryan *et al*. mapped the cell-type–resolved transcriptome (24), and Sophia Doll *et al*. presented the region- and cell-type–resolved proteome of the human heart (25). However, the interplay between different cell-types and their roles in various heart regions, as well as the transcriptional hierarchy regulating these functions in disease, has not been fully characterized.

In this study, we used the physiological mouse heart as a model to investigate the molecular features of various regions and cell-types. We isolated four heart cell-types, CMs, CFs, ECs, and IMs, and conducted both proteomic and transcriptomic analyses to create a cell-type–resolved proteome of the mouse heart. Additionally, we profiled the proteomes of six major regions (LA, RA, LV, RV, CP, and PF) and achieved in-depth region-resolved mouse heart proteome. The multiomics integrated analysis revealed the features of each cell-type, and their crosstalk facilitated through ligand-receptor-transcription factor-downstream genes. To further demonstrate the application of this proteomic atlas, we analyzed heart tissues from patients with DCM, uncovering region-specific alterations in protein expression. Remarkably, we found that the retinoic acid signaling pathway was enriched in the LV of DCM tissues, suggesting a potential therapeutic target for heart failure. In angiotensin II (Ang II)–stimulated neonatal rat cardiomyocyte (NRCM) hypertrophy and transverse aorta constriction (TAC)-induced heart failure mice model, all-trans retinoic acid (atRA) effectively rescued cardiac hypertrophy, further supporting its protective role. This study provides a comprehensive proteomic network of the heart at cell-type– and region-resolved levels, offering valuable insights into both physiological and pathological states.

## Experimental Procedures

### Animals and Tissue Collection

Six- to eight-weeks old C57BL/6, specific pathogen-free male mice (weight 20–25 g) were housed in pathogen-free, temperature-controlled environment. All mice were maintained with free access to food and water. All procedures were approved by IACUC, Fudan University. Ethical review approval number 201802143 S was obtained from the Department of experimental animal science, Fudan University.

### Clinical Sample Information

Formalin-fixed and paraffin-embedded (FFPE) human heart tissue, along with patient clinical information, were obtained from Zhongshan Hospital Fudan University. Experienced pathologists, Yingyong Hou and Rongkui Luo from Zhongshan Hospital Fudan University, collected both DCM and adjacent unaffected myocardium (AUM) areas from FFPE samples. This study was conducted in compliance with the ethical standards of Helsinki Declaration II and approved by the Institution Review Board of Fudan University Zhongshan Hospital (B2019-200R).

### Mouse Heart Isolation and Cell-Type Evalutation

Six- to eight-weeks old C57BL/6, specific pathogen-free male mice were used for heart cell-type isolation. The langendorff method was performed according to Liao, R., et, al.’s protocol (26). Adult mouse heart was collected and digested on Langendorff equipment using retrograde aortic perfusion with collagenase buffer (0.5 mg/ml collagenase II, 0.5 mg/ml collagenase IV, 0.05 mg/ml protease XIV). The resulting cell mixture was filtered through a 100 μm cell strainer–sterilized (SPL Singapore, Cat.No.93100) and centrifuged at 30*g* for 3 min at 4 °C to enrich for CMs. Nonmyocytes (CFs, ECs, IMs) were retained in the supernatant. We then stained the cell mixture with phenotypic markers and purified specific cell populations by fluorescence-activated cell sorting (FACS). Specifically, APC-conjugated-CD45 (Miltenyi Biotec, Cat.No.130-102-783), PE-conjugated-CD90 (BD Pharmingen, Cat.No.553014), and APC-conjugated-CD146 (Miltenyi Biotec, Cat.No.130-102-846) were used to label IMs, CFs and ECs, respectively. We purified cell populations for two separate measurements as follows, APC-conjugated-CD45 (IMs) and PE-conjugated-CD90 (CFs) sorted by one experiment and APC-conjugated-CD146 (ECs) sorted solely. All the data were acquired with BD influx and were analyzed with Flowjo 10.0.7.

### Cardiomyocytes Culture *In Vitro*

Primary CMs were cultured in M199 medium (Gibco, Cat.No.31100035) supplemented with 0.1% bovine serum albumin (BSA, Sigma, Cat.No.B2064), 1x ITS liquid media supplement (Sigma, Cat.No.I3146), 10 mmol/L 2,3-butanedione monoxime (Sigma, Cat.No.B0753), 1x chemically defined lipid concentrate (Gibco, Cat.No.11905031), and 1x Penicillin-Streptomycin (Gibco, Cat.No.15140122), at 37 °C and 5% CO_2_ in laminin-coated plates (Thermo Fisher Scientific, 23017-15) for 9 days.

### Cell and Animal Models

NRCMs were isolated from 1 to 3 days old Sprague-Dawley rats and cultured in Dulbecco's Modified Eagle Medium/Nutrient Mixture F-12 (DMEM/F12) (Invitrogen, 11330032) with 10% fetal bovine serum for 24 h. Then medium was changed into serum-free DMEM/F12 for 24 h. One micromolar of Angiotensin II (MCE, HY- 13948) treated cells individually or treated cells with 1 μM atRA (Sigma, R2625) collectively for 24 h. After 24 h incubation, cardiomyocytes were collected and lysed for further quantitative real-time PCR (qRT-PCR).

In the pressure overload hypertrophy model induced by TAC, WT C57BL/6 mice were randomly divided into sham group, TAC group, atRA group, TAC + atRA group. Fresh atRA was dissolved in corn oil each day to limit spontaneous conversion to its 9- and 13-cis stereoisomers. Mice in the sham and TAC groups were administered either atRA (60 mg/kg/day) or corn oil (vehicle control) by gavage for 4 weeks.

### Histological Analysis

Heart tissues were fixed in 10% formalin for histological and histomorphometric assessment, embedded in paraffin, then sectioned at 3 μm thickness (Leica, RM2235). After deparaffinization and rehydration, 3 μm longitudinal sections were stained with hematoxylin solution for 5 min followed by five dips in 1% acid ethanol (1% HCl in 70% ethanol) and then rinsed in distilled water. Sections were stained with H&E or wheat germ agglutinin (WGA) (Invitrogen) to measure myocyte cross-sectional areas. H&E-stained slides were examined and photographed using a digital whole Slide Scanner (Leica, Aperio AT2). WGA-stained slides were analyzed with a Nikon TS100 fluorescence microscope and myocyte cross-sectional areas were calculated using ImageJ software.

### Masson Staining

Paraffin-embedded tissues were sectioned at 3 μm thickness, deparaffinized with xylene, and then rehydrated with different graded alcohols. Trichrome II Blue staining kit (860-013; Ventana Medical Systems, Inc.) was used on the BenchMark Special Stains automated slide staining instruments. Trichrome II Blue staining kit is a modification of Masson’s Trichrome Stain. Briefly, heart sections were incubated with Bouin’s solution to intensify the final coloration. Cytoplasm and muscle were stained with Trichrome Red, containing Biebrich scarlet and acid fuchsin, while nuclei were stained with iron hematoxylin. After application of Trichrome Mordant, the collagen was stained with Trichrome Blue II, which contains aniline blue. Trichrome Clarifier, an acetic acid solution, was applied to create a more delicate and transparent shade of color in the heart tissue section. The mounted slides were then examined and photographed using a digital whole Slide Scanner (Leica, Aperio AT2).

### Immunofluorescence Analysis

NRCMs were fixed with 4% paraformaldehyde for 15 min, permeabilized in 0.1% Triton X-100 (Sigma) for 10 min, blocked in 3% BSA for 1 h, and then incubated with primary antibodies against α-actinin (1:300; Sigma) overnight at 4 °C. The cells were washed with PBS and detected by Alexa Fluor 594-conjugated secondary antibodies. Nuclei were stained with Hoechst33258 (Sigma). Immunofluorescence images were captured using a Nikon TS100 fluorescence microscope, and myocyte cross-sectional areas were calculated with ImageJ.

### Quantitative Real-Time PCR

Total RNA was extracted from the heart LV tissue or NRCMs using TRIzol (Invitrogen) according to the manufacturer’s instructions. Reverse transcription of purified RNA was obtained by RevertAid RT Reverse Transcription Kit (Thermo Fisher Scientific). The quantification of gene transcripts was performed by quantitative PCR using SYBR Premix Ex Taq (TAKARA). All values were normalized to the level of Gapdh mRNA. The RT-qPCR primers are listed in Supplemental Table S6.

### Western Blotting

Heart tissues were minced, homogenized, and digested in tissue lysis buffer (with protease and phosphatase inhibitors; Thermo Fisher Scientific). Denatured protein was loaded onto polyacrylamide gels, and after electrophoresis, proteins were transferred to PVDF membranes (Millipore), then were blocked in 5% nonfat milk for 1.5 h at room temperature. Incubation of primary antibodies was done overnight at 4 °C in 3% BSA on TBS with 0.1% Tween-20. Primary antibodies were as follows: mouse anti-GAPDH (Kangchen, KC-5G5, 1:5000), rabbit anti-Rxra (ABclonal, A15242, 1:1000), rabbit anti-Rxrb (ABclonal, 18,119, 1:1000). Subsequently, the membrane was incubated with a horseradish peroxidase–conjugated secondary antibody. Bands were visualized after activation with ECL (Thermo Fisher Scientific) for detection of protein expression.

### Echocardiography Measure

Echocardiography was performed on lightly anesthetized mice-given isoflurane (induction with 1% isoflurane 100% O2 and maintained with 0.2–0.25% isoflurane 100% O2) to maintain high cardiac frequency. Transthoracic echocardiography (Vevo 2100; Visual Sonics) with a 25-MHz imaging transducer was performed on anesthetized animals with measurement of LVEF, LVFS, LVDs, and other parameters as previously reported (27).

### Protein Sample Preparation

#### Whole-Cell and Whole-Tissue Protein Extractions

To extract proteins from whole cells and tissues, aliquots of CMs, CFs, ECs, IMs, or 0.1 g heart tissue were lysed with 400 μl urea lysis buffer (8 M urea, 100 mM Tris–HCl pH 8.0); 4 μl protease inhibitor (PierceTM, Thermo Fisher Scientific) was added to protect protein from degradation and protein concentrations were measured using Bradford method (Eppendorf Biospectrometer).

### Protein Exaction from FFPE Samples

FFPE samples were collected dewaxed and lysed in a buffer consisting of 2% deoxycholate, 10 mM Tris (2-carboxyethyl) phosphine, 40 mM chloroacetamide, 100 mM Tris–HCl at pH 8.8, and 1 mM PMSF at 99 °C for 30 min, as we previously described (28). The crude exact was then clarified by centrifugation at 16,000*g* for 10 min and the supernatants were loaded into 10 kD Microcon filtration devices (Millipore) and centrifuged at 12,000*g* for 20 min and washed twice with Urea lysis buffer (8 M Urea, 100 mM Tris–HCl pH 8.0), twice with 50 mM NH_4_HCO_3_. Proteins were digested using trypsin at an enzyme-to-protein ratio of 1:25 overnight at 37 °C. Peptides were extracted and dried using a SpeedVac (Eppendorf).

### catTFRE Pulldown and Trypsin Digestion

We selected consensus transcription factor response elements (TFREs) for different transcription factor (TF) families using the JASPAR database. For constructing the catTFRE, 100 selected TFREs were arranged in two tandem copies with a three-nucleotide spacer, yielding a total DNA length of 2.8 kb. Nuclear extracts (100 μg) were then used for the catTFRE pulldown assay. The 2.8 kb catTFRE DNA and biotinylated primers were synthesized by Genscript. For the pulldown, 1 pmol of biotinylated DNA, pre-bound to Dynabeads (M280 streptavidin, Thermo Fisher Scientific), was incubated with nuclear extracts at 4 °C for 2 h. Following incubation, beads were washed twice with NETN buffer (100 mM NaCl, 20 mM Tris–HCl, 0.5 mM EDTA, and 0.5% [vol/vol] Nonidet P-40) and PBS. The beads were then suspended in 100 μl of 50 mM ABC and digested with trypsin for 12 h, with peptides subsequently extracted using 50% acetonitrile (ACN) + 0.1% formic acid (FA) and dried under vacuum for mass spectrometry analysis.

### Protein Trypsin Digestion and First Dimension RPLC

For whole-cell and whole-tissue proteomes, 100 μg of proteins were digested using the FASP protocol. Protein samples were supplemented with 1 M DTT to a final concentration of 5 mM and incubated for 30 min at 56 °C. Iodoacetamide was added to a final concentration of 20 mM, and samples were incubated in the dark at room temperature for 30 min. Then samples were added 5 mM final concentration of DTT and kept in dark for another 15 min. After these procedures, protein samples were loaded into 10 kD Microcon filtration devices (Millipore) and centrifuged at 12,000*g* for 20 min and washed twice with Urea lysis buffer (8 M Urea, 100 mM Tris–HCl pH 8.0), twice with 50 mM NH_4_HCO_3_. Then the samples were digested using trypsin at an enzyme-to-protein ratio of 1:25 overnight at 37 °C. Peptides were extracted and dried using a SpeedVac (Eppendorf).

Proteins from cell-types– and region-resolved mouse samples were digested into peptides and then performed first dimension RPLC before LC-MS/MS. The dried peptides were loaded into a homemade Durashell RP column (2 mg packing (3 μm, 150 Ǻ, Agela) in a 200 μl tip), then eluted sequentially with nine gradient elution buffer which contains mobile phases A (2% ACN, adjusted pH to 10.0 using NH_3_.H_2_O) and 6%, 9%, 12%, 15%, 18%, 21%, 25%, 30%, 35% mobile phase B (98% ACN, adjusted pH to 10.0 using NH_3_.H_2_O). The nine fractions then were combined into six groups (6% + 25%, 9% + 30%, 12% + 35%, 15%, 18%, 21%) and dried under vacuum for subsequential MS analysis, as we previously described (29). Peptides redissolved in solvent A (0.1% FA in water) and separated on a 150-μm-inner-diameter column with a length of 15 cm (1.9-μm ReproSil-Pur C18-AQ beads, Dr Maisch GmbH) over a 75 min gradient at a constant flow rate of 600 nl/min.

Proteins from human DCM samples, adult mouse cardiac myocytes cultured *in vitro*, and atRA-treated mice *in vivo* were digested into peptides and then performed first dimension RPLC before LC-MS/MS. Peptide samples were loaded into a homemade trap column (100 μm × 2 cm; pore size, 120 Å; particle size, 3 μm; SunChrom) and then separated with a gradient of 4%–100% mobile phase B (80% ACN and 0.1% FA) at a flow rate of 600 nl/min for 150 min gradient by a homemade silica microcolumn (150 μm × 30 cm; pore size, 120 Å; particle size, 1.9 μm; SunChrom).

### LC-MS/MS Analysis

Peptides from catTFRE pulldown and peptides from cell-types– and region-resolved mouse samples, human samples were detected by Orbitrap Fusion Lumos (Thermo Fisher Scientific). Peptides from NRCM cell samples were detected by Q Exactive HFX (Thermo Fisher Scientific).

Orbitrap Fusion Lumos LC-MS/MS analyses were performed on an Easy-nLC 1000 liquid chromatography system (Thermo Fisher Scientific) coupled to an Orbitrap Fusion Lumos via a nano-electrospray ion source (Thermo Fisher Scientific). Fractions from the first dimension RPLC were dissolved with loading buffer (5% methanol and 0.2% FA) and loaded onto a 360 μm I.D. × 2 cm, C18 trap column at a maximum pressure 280 bar with 12 μl solvent A (0.1% FA in water). Peptides were separated on 150 μm I.D. × 14 cm column (C18, 1.9 μm, 120 Ǻ, Dr Maisch GmbH) with a series of adjusted linear gradients according to the hydrophobicity of fractions with a flow rate of 600 nl/min for 75 min or 150 min gradient. The MS analysis was performed in a data-dependent manner with full scans (m/z 350–1500) acquired using an Orbitrap mass analyzer at a mass resolution of 120,000 at m/z 200. The top speed data-dependent mode was selected for fragmentation in the HCD cell at normalized collision energy of 30%, and then fragment ions were transferred into the ion trap analyzer. The automatic gain control for full MS was set to 5e5 and that for MS/MS was set to 1e4, with maximum ion injection times of 50 ms and 10 ms, respectively. The dynamic exclusion of previously acquired precursor ions was enabled at 45 s, cycle time is 1s.

Q Exactive HFX LC-MS/MS analyses were performed on an Easy-nLC 1000 liquid chromatography system (Thermo Fisher Scientific) coupled to a Q Exactive HFX via a nano-electrospray ion source (Thermo Fisher Scientific). The peptides were dissolved with 10 μl loading buffer (5% methanol and 0.2% FA), and 5 μl was loaded onto a 360 μm I.D. × 2 cm, C18 trap column at a maximum pressure 280 bar with 12 μl solvent A (0.1% FA in water). Peptides were separated on 150 μm I.D. × 14 cm column (C18, 1.9 μm, 120 Ǻ, Dr Maisch GmbH) with a linear 5 to 35% mobile phase B (ACN and 0.1% FA) at 600 nl/min for 75 min. The MS analysis was performed in a data-dependent manner with full scans (m/z 300–1400) acquired using an Orbitrap mass analyzer at a mass resolution of 120,000 at m/z 400. The top 20 precursor ions were selected for fragmentation in the HCD cell at normalized collision energy of 32%, and then fragment ions were transferred into the Orbitrap analyzer operating at a resolution of 7500 at m/z 400. The AGC for full MS was set to 3e6 and that for MS/MS was set to 5e4, with maximum ion injection times of 80 ms and 20 ms, respectively. The dynamic exclusion of previously acquired precursor ions was enabled at 12 s.

### Peptide and Protein Identification

MS raw files were processed with the “Firmiana” (a one-stop proteomic cloud platform and utilized Mascot 2.7 search engine) (30) against the mouse RefSeq protein database (released on 04-07-2013; 27,414 entries) and the human RefSeq protein database (released on 04-07-2013; 32,016 entries) in the National Center for Biotechnology Information. The maximum number of missed cleavages was set to 2. A mass tolerance of 20 ppm for precursor ions and 0.5 Da for product ions were allowed. The fixed modification was cysteine carbamidomethylation and the variable modifications were N-acetylation and oxidation of methionine. For the quality control of proteins identification, the target-decoy–based strategy was applied to control the false discovery rate (FDR) of both peptide and protein, which was lower than 1%. Percolator was used to obtain the probability value (q-value), validating the FDR (measured by the decoy hits) of every peptide-spectrum match was lower than 1%. Then all the peptide lengths shorter than seven amino acids were removed. The cutoff ion score for peptide identification was 20. All the peptide-spectrum matchs in all fractions were combined for protein quality control, which was a more stringent quality control strategy. The q-values of both target and decoy peptide sequences were dynamically increased until the corresponding protein FDR was less than 1% employing the parsimony principle. Finally, to reduce the false positive rate, the proteins with at least two unique peptides were selected for further investigation.

### Label-Free–Based MS Quantification of Proteins

The one-stop proteomic cloud platform “Firmiana” (30) was further employed for protein quantification. Identification results and the raw data from mzXML file were loaded. Then for each identified peptide, the extracted-ion chromatogram was extracted by searching against the MS1 based on its identification information, and the abundance was estimated by calculating the area under the extracted extracted-ion chromatogram curve (AUC). For protein abundance calculation, the nonredundant peptide list was used to assemble proteins following the parsimony principle. Then, the protein abundances are estimated with a traditional label-free, intensity-based absolute quantification (iBAQ) algorithm, which divided the protein abundance (derived from identified peptides’ intensities) by the number of theoretically observable peptides. Then the fraction of total (FOT), a relative quantification value which was defined as a protein’s iBAQ divided by the total iBAQ of all identified proteins in one experiment, was calculated as the normalized abundance of a particular protein among experiments. Finally, the FOT was further multiplied by 10^5^ for the ease of presentation. Missing values were imputed with the smallest value across the matrix.

### RNA extraction

Total RNA was extracted from cell-types and heart tissue using TRIzol Reagent according to the manufacturer’s instructions. Then RNA quality was determined by 5300 Bioanalyser (Agilent) and quantified using the ND-2000 (NanoDrop Technologies). Only high-quality RNA sample was used to construct sequencing library.

### Library Preparation and Sequencing

RNA purification, reverse transcription, library construction, and sequencing were performed according to the manufacturer’s instructions. RNA-seq transcriptome library was prepared following Illumina Stranded mRNA Prep, Ligation using 1 μg of total RNA. Shortly, messenger RNA was isolated according to polyA selection method by oligo (dT) beads and then fragmented by fragmentation buffer firstly. Secondly double-stranded cDNA was synthesized using a SuperScript double-stranded cDNA synthesis kit (Invitrogen) with random hexamer primers. Then the synthesized cDNA was subjected to end-repair, phosphorylation, and adapter addition according to library construction protocol. Libraries were size selected for cDNA target fragments of 300 bp on 2% low range ultra agarose followed by PCR amplified using Phusion DNA polymerase (NEB) for 15 PCR cycles. After quantified by Qubit 4.0, the sequencing library was performed on NovaSeq X Plus platform (PE150) using NovaSeq Reagent kit.

### Quality Control and Read Mapping

The raw paired end reads were trimmed and quality controlled by fastp (31) with default parameters. Then clean reads were separately aligned to reference genome with orientation mode using HISAT2 (32) software. The mapped reads of each sample were assembled by StringTie (33) in a reference-based approach.

### TF Classification

Proteins identified by TFRE pulldown were categorized into DNA-binding proteins, transcription factors (TFs), and transcription co-regulators (TCs). We extracted DNA-binding proteins by filtering the genes’ description “DNA-binding.” And we extracted TFs and TCs by filtering the gene symbols, using the gene symbols list of TFs and TCs, from public databases described in previous studies (34).

### Statistical Analysis for RT-PCR and Echocardiography Data

Data are presented as mean ± SEM. When related RT-PCR datasets, statistical significance was analyzed by using unpaired Student’s *t* test or ANOVA, followed by Bonferroni’s multiple comparisons test as appropriate. Echocardiographic data were analyzed using two-way ANOVA followed by Tukey’s multiple comparisons test. All statistical analyses were performed using Graphpad Prism software (version 7).

### Experimental Design and Statistical Rationale

Proteomic profiling was conducted on cell-type resolved (CM, CF, EC, and IM) and region resolved (LA, LV, RA, RV, CP, and PF) heart samples, each with three biological replicates. Proteins that expressed in one cell-type twice greater than the geometric mean in other three cell-types were defined as the cell-type–enhanced proteins. We performed proteome in four regions (LA, LV, RA, and RV) with AUM area and DCM area from 12 dilated cardiomyopathy samples. Compared AUM area with DCM area, unpaired *t* test was used and statistical significance was considered at a *p* value < 0.05. Given sample size limitation, we chose to present the data using *p* value < 0.05 to avoid potential loss of meaningful biological information.

### Bioinformatics and Statistical Analysis for MS Data

Hierarchical clustering was performed using the pheatmap (Pretty Heatmaps) function in the R package (pheatmap, version 1.0.8). Gene Ontology (GO) and Kyoto Encyclopedia of Genes and Genomes (KEGG) pathway enrichment analysis was determined using the Database for Annotation, Visualization and Integrated Discovery (DAVID) (35) with Fisher’s exact test.

### The Calculation of Overexpressed Genes Between Protein and RNA Level

Figure 3*G* shows that linear fitting was done according to the expression levels of protein (Log_2_FOT) and RNA (Log_2_FPKM), and the middle line was obtained, which was used as the benchmark for the corresponding relationship between the expression levels of protein and RNA. The deviation degree (also called as intercept) between each pair of protein and RNA was calculated according to the slope of the baseline. The first 5% were selected as protein overexpressed and the last 5% as RNA overexpressed according to the rank from largest to smallest intercept. The top line is the intercept size of the sorted number in first 5%, and the bottom line is the last 5%. The red dots represent genes overrepresented in the proteome and the blue dots represent genes overrepresented in transcriptome, with enriched GO terms showed beside.

### WGCNA Consensus Network Construction and Module Enrichment Analysis

The general framework of weighted gene co-expression network analysis (WGCNA) has been described in previous publication (36) and has been packaged into a user-friendly R library. In short, we constructed a signed network for the proteins detected in the four regions of heart (LV, LA, RA, and RV). Seven modules were assigned, and initial module assignments were determined by using a dynamic tree-cutting algorithm (cutreeHybrid, using default parameters except deepSplit = 4, cutHeight = 0.999, minClusterSize = 30, and pamStage = FALSE).

### Ligand-Receptor-TF-TG Network

The ligand–receptor interactions were downloaded from the DLRP and IUPHAR databases. Only the interactions between cell-type–enriched ligands and receptors that were identified in the corresponding myocytes with significantly enriched downstream KEGG signaling pathways were selected (hypergeometric test, *p*-value <0.05). Cell-type–enriched ligands were identified using the ratio of a protein expression level in a particular cell-type to the average level in the four cell-types (at least 1.5-fold). Enriched signaling pathways were defined as those with cell-type–expressed TFs and significantly enriched pathway nodes within the receptor-TF branch that could be detected in the proteomic profile (hypergeometric test, *p*-value <0.05). The network among TFs and target genes (TGs) was obtained from CellNet (37). Data visualization was done with Cytoscape v 3.3.0.

## Results

### Proteome Profiling of Four Cell-Types of Mouse Heart

**Fig. 1:**
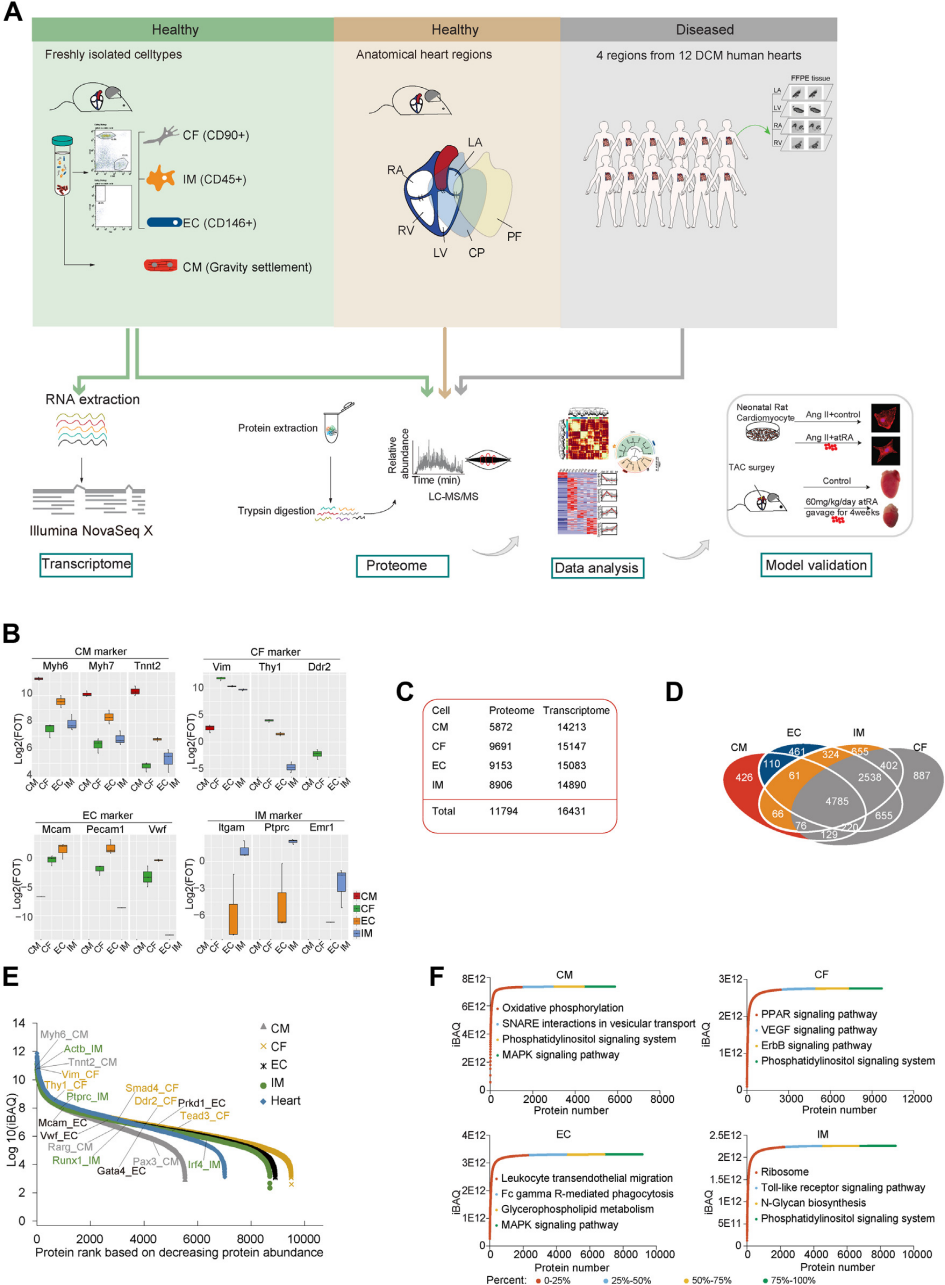
**Proteome and transcriptome of four major heart cell-types.***A*, schematic illustration of the experimental workflow. Four major heart cell-types (CM-cardiac myocyte; CF-cardiac fibroblast; EC-endothelial cell; IM-immune cell) were isolated by flow cytometer or gravity settlement for proteomic and transcriptomic profiling. Six anatomical regions were analyzed on a high-resolution mass spectrometer. Four regions from 12 dilated cardiomyopathy samples were analyzed at the protein level. *B*, the box plots indicate the expression level of well described markers for CMs, CFs, ECs, and IMs. *C*, identification of gene products (GP) numbers at the protein and mRNA levels for major heart cell-types and intact heart tissue. *D*, Venn diagram of the identified GP numbers at the protein level among four cell-types. *E*, dynamic range of the proteome of heart cell-types, based on protein abundance (Log10(iBAQ)). The different colors represent high, intermediate, and low abundant proteins in four cell-types and heart tissue. *F*, cumulative protein mass from the highest to the lowest abundance proteins for indicated cell-types along with enriched GOBP pathways. The color represents percentage of these proteins that showed cell-type–enriched expression.

To map the molecular landscape of the cell-type– and region-resolved heart proteome, we performed transcriptome and proteome profiling, integrating multiomics as illustrated in the experimental workflow (Fig. 1*A*). Mouse hearts were digested using the langendorff method (26) for retrograde aortic perfusion, and the markers CD90 (CFs), CD146 (ECs), CD45 (IMs) were chosen for FACS (38), while CMs were purified by sequential gravity settlement as previously described (38). Smooth muscle cells were not analyzed due to the lack of surface markers for FACS, as common markers like α-SMA and ACTA2 mainly label cytoskeleton proteins (25, 38). The yields for CMs, CFs, ECs, and IMs were approximately 1e7, 2.1e5, 2.8e5, and 1.9e5 cells per mouse, respectively. FACS analysis confirmed high purities of CFs (95.6%), ECs (96.4%), IMs (99.3%) (Supplemental Fig. S1*A*). We applied microscopic count to check the purity and vitality of CMs, and as shown in Supplemental Fig. S1*A*, rod shaped myocytes represented 94.5% of the cells from the average of 10 repeats independent counts. Proteomic analysis of cell-type–specific proteins confirmed the high purity of isolated cell populations, with key markers like myosin heavy chain 6 (Myh6), myosin heavy chain 7 (Myh7), and troponin T2 (Tnnt2) enriched in CMs, and Thy-1 cell surface antigen (Thy1), melanoma cell adhesion molecule (Mcam), and protein tyrosine phosphatase receptor type C (Ptprc) elevated in CFs, ECs, and IMs, respectively (Fig. 1*B*).

We employed the Fast-seq approach for proteomic profiling (39, 40), using three biological replicates for each cell-type and region. In addition to the four major cell-types, we also collected whole-heart tissue for proteomic analysis. As a result, we identified the proteomes of each cell-type in the range of 5872 proteins in CMs to 9691 proteins in CFs (Fig. 1, *C* and *D*, Supplemental Fig. S1, *B* and *C*, and Supplemental Table S1) and a total of 11,794 proteins. Biological replicates exhibited high correlations in protein abundance in the same cell-type (R > 0.86), and proteome quantification across 18 experiments was highly consistent, as indicated by comparable median values (Supplemental Fig. S1, *D* and *E*).

Principal component analysis (PCA) revealed a closer correlation between CMs and the whole-heart tissue, suggesting that the proteome pattern and biological process of the heart are mainly represented by CMs (Supplemental Fig. S1*F*). We used the iBAQ algorithm (41) for protein abundance quantification, and iBAQ values were normalized to the FOT (Experimental procedures). The dynamic range of relative protein abundance in the cell-type–resolved mouse heart proteome spanned nearly nine orders of magnitude (Fig. 1*E*). Our dataset achieved deep proteome coverage, identifying both highly abundant proteins (Myh6, Tnnt2, Vim, Vwf) and low-abundance proteins, including transcription factor Gata4, Pax3, Tead3, and Irf4 (Fig. 1*E*). The cumulative abundance curve reflected the bioprocess features of different cell-types, with oxidative phosphorylation ranked as the top bioprocess in CMs, while CFs showed higher enrichment in PPAR and VEGF signaling pathways compared to other cell-types (Fig. 1*F*, Supplemental Table S1).

### Proteome Features of the Four Heart Cell-Types

**Fig. 2:**
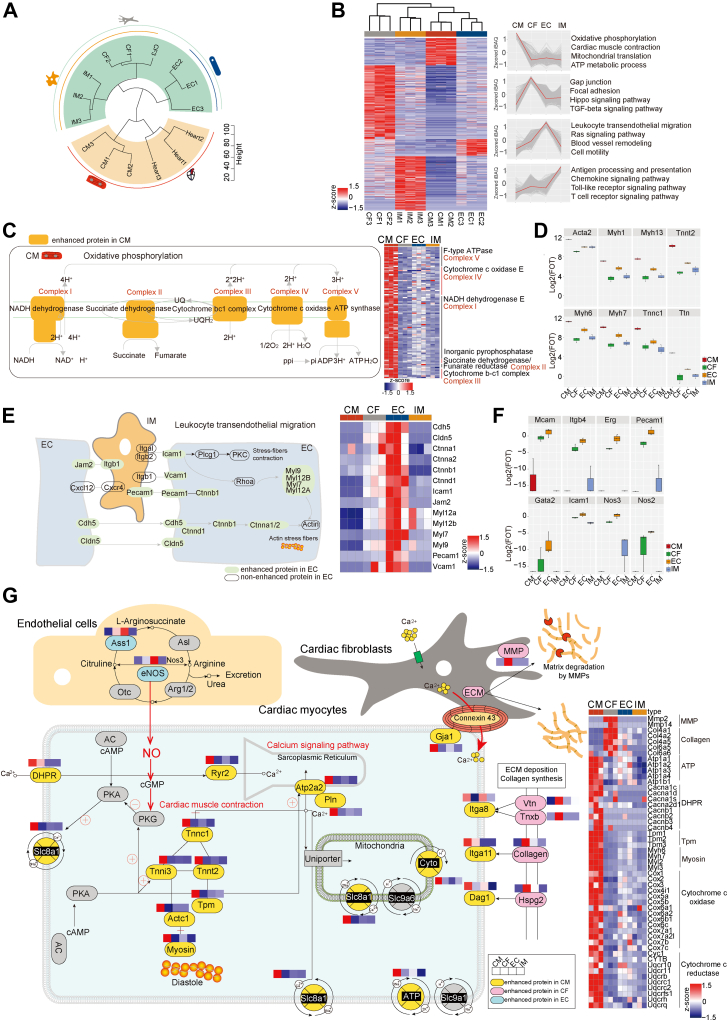
**Proteome features of the four major heart cell-types.***A*, the hierarchical clustering of the major heart cell-types and whole heart with euclidean distance. *B*, clustering heatmaps show the cell-type–enhanced proteins’ expression pattern in four major heart cell-types. The color bar indicates z-scored iBAQ. The top GOBP/KEGG terms enriched by each cluster are shown on the *right*. *C–F*, the protein expressed patterns of oxidative phosphorylation, leukocyte transendothelial migration in the proteome of CMs (*C*) and ECs (*E*). The heatmaps represent the expression patterns of critical proteins participate in the pathway. The color bar of heatmap indicates normalized z-scored iBAQ. The boxplots show cell-type–enhanced proteins in CMs (*D*) and ECs (*F*), including transcription factors and proteins functioned in cell-type. *G*, the protein expressed patterns of the arginine biosynthesis (ECs), ECM deposition (CFs), calcium signaling pathway, and cardiac muscle contraction (CMs) in the proteome of the major heart cell-types. The color bar indicates normalized z-scored iBAQ. Clustering heatmaps show protein families of CFs and CMs on the *right*.

Hierarchical clustering analysis grouped CMs with whole-heart tissue, while CFs, ECs, and IMs formed a separate cluster, highlighting the proteomic similarity between CMs and the whole-heart tissue (Fig. 2*A*). Proteins expressed at least two-fold higher in 1 cell-type than the geometric mean of the other three were classified as cell-type–enhanced proteins. Gene Ontology Biological Processes (GOBP) and KEGG enrichment analyses of cell-type–enhanced proteins revealed a strong correlation between proteome features and the physiological functions. For instance, CMs were dominant in oxidation phosphorylation and ATP metabolic processes, CFs were enriched in TGF-beta and hippo signaling pathways, ECs were associated with leukocyte transendothelial migration, and IMs were specific to Toll-like receptor and T cell receptor signaling pathways (Fig. 2*B*, Supplemental Table S1).

We further explored representative dominant pathways across the four cell-types. Components of the respiratory chain complexes I, II, III, IV, and V were overrepresented in CMs, along with myosin family proteins (Myh1, Myh6, Myh7), which play key roles in muscle contraction (Fig. 2, *C* and *D*). TGF-beta signaling, including Smad and Smurf families, was highly expressed in CFs (Supplemental Fig. S2, *A* and *B*), while leukocyte transendothelial migration pathway was dominant in ECs (Fig. 2, *E* and *F*). In IMs, components of traditional immune pathway, such as the Tlr, Irf, Ifn families, were highly regulated (Supplemental Fig. S2, *C* and *D*).

The protein abundance patterns of complement and coagulation cascades showed complementary expression across cell-types. For instance, IMs expressed coagulation factors F5 and F8, along with thrombin inhibitors such as Serpinc1, Serpind1, and Serpinf2 but exhibited low levels of Plau and Plat, key enzymes in plasmin activation. Interestingly, while F8 was expressed in IMs, VWF was exclusively expressed in ECs (Supplemental Fig. S2*E*). This differential expression pattern suggests a division of labor between cell-types in maintaining cardiac homeostasis. We also examined crosstalk among cell-types by screening protein expression in pathways such as arginine biosynthesis, muscle contraction, and calcium ion transduction. In arginine biosynthesis, nitric oxide synthase (NOS) including iNOS (Nos2) and eNOS (Nos3) were dominantly expressed in ECs (Fig. 2*F*), while proteins participated in NO-favored pathways, including cardiac muscle contraction, were primarily expressed in CMs (Fig. 2*G*). This suggests that ECs play a key role in supporting CM function. Additionally, gap junction proteins like Connexin 43 (Gja1), known for transporting Ca^2+^, were predominantly expressed in CMs, indicating the possibility that Ca^2+^ transduction from other cells to CMs to maintain efficient contraction. MMP family proteins, involved in ECM maintenance, were highly expressed in CFs, highlighting their role in providing structural support to CMs (Fig. 2*G*).

### Comparison Between Proteome and Transcriptome

**Fig. 3:**
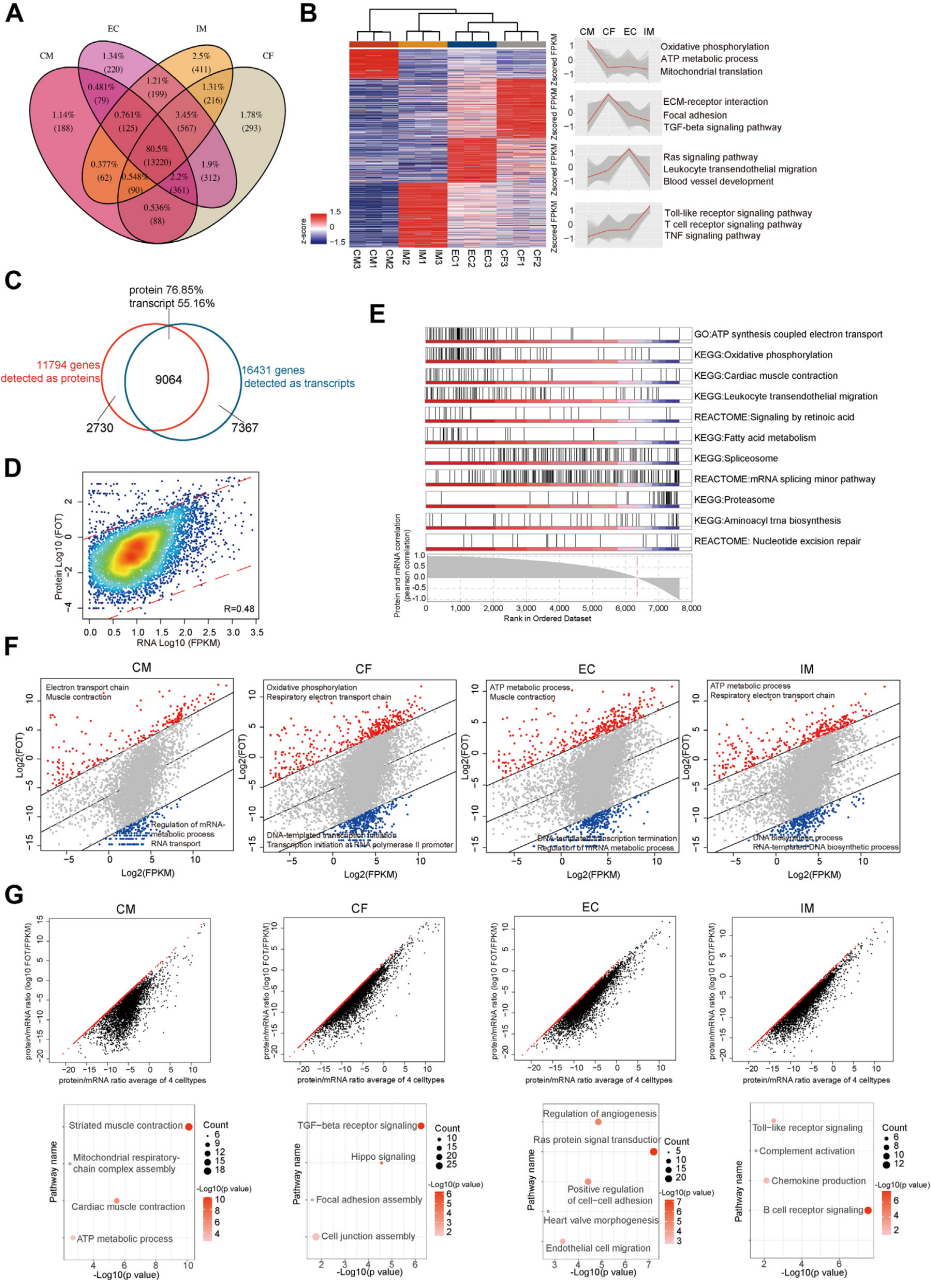
**Transcriptome feature of the four heart cell-types.***A*, Venn diagram showing the identified gene numbers at the transcriptome level among the four major heart cell-types. *B*, clustering heatmaps show the cell-type–enriched genes’ transcription pattern in four major heart cell-types. The color bar indicates normalized z-scored FPKM. The top GOBP/KEGG terms enriched by each cluster are shown on the *right*. *C*, Venn diagram of the identified gene numbers at the protein and mRNA level for the whole four cell-types. *D*, the correlation between protein and mRNA intensities for all four cell-types. R is the Spearman correlation coefficient. *E*, the enriched GOBP, reactome, and KEGG pathways by Gene set enrichment analysis (GSEA). The curve below represents the Pearson Correlation between protein and transcript expression value of every gene among four cell-types. Each *black* line represents a gene from the GO pathway on the *right*, with its position on the x-axis determined by its Pearson Correlation coefficient shown on the *bottom*. *F*, the scatter plots of FOT intensities of proteome versus FPKM values of transcriptome in four cell-types. The *red* dots represent genes overrepresented in the proteome and the *blue* dots represent genes overrepresented in transcriptome, with enriched GO terms shown in the corner. *G*, the scatter plots (*top*) show the average ratio of protein and RNA in four cell-types on the x-axis and the ratio of protein and RNA in one cell-type (CM, CF, EC, or IM) on the y-axis. If the average ratio in four cell-types (x-axis) is three times higher than the average ratio in one cell-type (y-axis), the *red* dot is shown. The genes in *red* dot were used for GOBP/KEGG enrichment by DAVID software and calculated by Fisher’s exact test. The bubble plots (*bottom*) show the GOBP/KEGG-enriched pathways.

To compare gene expression at the protein and mRNA levels, we profiled the transcriptome of the heart’s major cell-types using next-generation sequencing, identifying a total of 16,431 transcripts, ranging from 14,213 transcripts in CMs to 15,147 transcripts in CFs (Figs. 1*C* and 3*A*). Cell-type–enhanced genes were defined based on the mRNA level, and GOBP/KEGG enrichment revealed similar patterns for both mRNA and protein, with oxidation phosphorylation process dominant in CMs and Toll-like receptor and TNF signaling pathways enriched in IMs, indicating consistency between gene expression and its functional output (Fig. 3*B*, Supplemental Table S2).

Nevertheless, despite the general similarity between transcriptome and proteome data, there were notable differences between the two: 1) the overlap between protein and mRNA profiles was approximately 76.85% at the proteome level and 55.16% at the transcriptome level (Fig. 3*C*), with a medium overlap between mRNA and protein expression, as reported in previous studies (22, 42) (Supplemental Fig. S3*A*); 2) the median correlation between total gene expression abundances at the transcriptome and proteome levels was 0.48 (Spearman correlation coefficient) and correlation among CMs, CFs, ECs, and IMs was 0.53, 0.49, 0.46, and 0.49, respectively (Fig. 3*D*, Supplemental Fig. S3, *B*–*E*); 3) gene set enrichment analysis revealed that genes with a strong correlation between transcript and protein levels were primarily involved in ATP synthesis, oxidative phosphorylation, and cardiac muscle contraction, whereas genes with weaker correlations were enriched in proteasome activity and nucleotide excision repair (Fig. 3*E*, Supplemental Table S2).

Further analyses of protein and mRNA expression within each of the four cell-types revealed that the high abundance proteins were enriched in ATP metabolism while the high abundance mRNAs were enriched in RNA process and DNA biosynthetic process (Fig. 3*F*, Supplemental Table S2). Additionally, a comparison of proteome and transcriptome patterns across the four cell-types showed that genes with a higher proteome-to-transcriptome ratio were associated with cell-type–specific biological functions. For instance, genes with a higher ratio in CMs participated in cardiac muscle contraction, a key function of CMs (Fig. 3*G*). These comparisons highlight the biases in the translation of protein from mRNA, especially in relation to cell-type–specific functional features (Fig. 3*G*, Supplemental Table S2).

### Cell-Type–Specific Transcription Factor Panels and Their Correlation with Major Cellular Processes

**Fig. 4:**
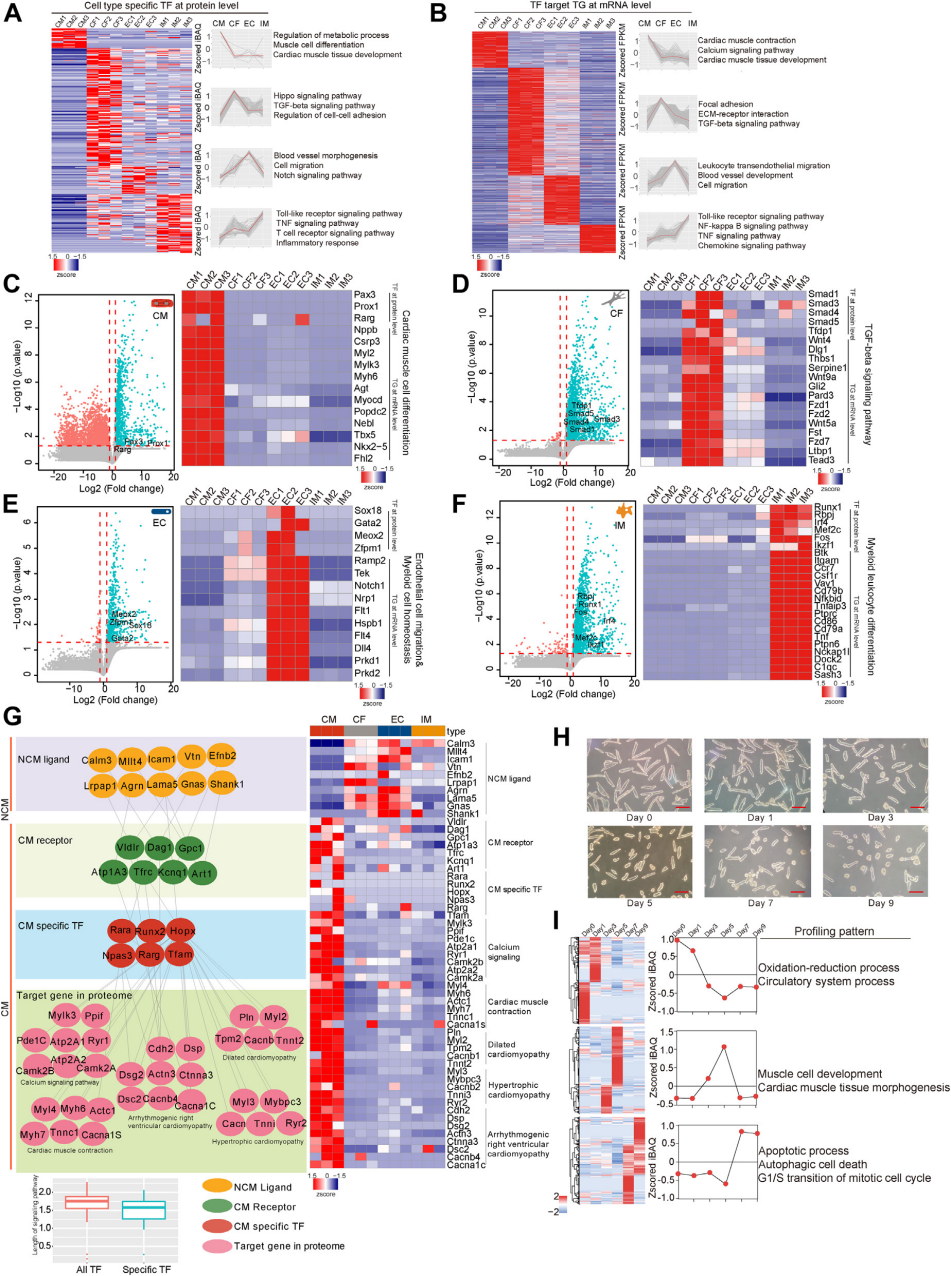
**The transcription factors centered proteome for the four major heart cell-types.***A* and *B*, clustering heatmaps show the cell-type–specific transcription factors at the protein level and their target genes at the mRNA level in the four cell-types. The color bar indicates normalized z-scored iBAQ for TFs and normalized z-scored FPKM for TGs. Gene Ontology (GO) and Kyoto Encyclopedia of Genes and Genomes (KEGG) pathway enrichment analysis were performed using the DAVID with Fisher’s exact test. *C*–*F*, volcano plot of the *p* values versus the log2 protein abundance differences in indicated cell-type compared to other three cell-types. Significantly upregulated and downregulated proteins are highlighted in *green* and *red*, respectively. Heatmap on the *right* shows specific TF and TG for the indicated cell-types. *G*, network of the crosstalk between cardiac myocytes and nonmyocytes. *Orange* molecules indicate ligands, *green* indicate receptors, *red* indicate CM-specific TFs, and *pink* indicate TGs at protein level. The boxplot shows the distance comparison between all identified transcription factors in cardiac myocytes with nonmyocytes proteins and cardiac myocyte–specific transcription factors with noncardiac myocyte proteins. The heatmap shows the protein expression from the network. *H*, the images of cardiac myocytes cultured *in vitro* for 0, 1, 3, 5, 7, 9 days. Scale bars represent 100 μm. *I*, the profiling expression patterns of the three clusters proteins identified in the proteome for *in vitro*–cultured cardiac myocytes, with GO items enriched by each cluster of proteins on the *right*. The color bar indicates normalized z-scored iBAQ.

TFs play critical roles in regulating a variety of biological processes (43). Our study achieved deep coverage in heart proteome, which helped better describe TF patterns and their relationship with their downstream genes. We have detected 479 TFs in the mouse heart, a greater number than the 272 TFs detected in previous study (44), which used catTFRE enrichment (45). This demonstrates the advantage of cell-type–resolved organ proteome in detecting low-abundance proteins such as TFs. We applied the same criteria for determining cell-type–enhanced proteins to nominate the cell-type–specific TFs (ctsTFs) (at least two-fold greater than geometric mean in other three cell-types). To investigate the downstream targets of ctsTFs, we used the CellNet TF-TG database to screen out transcriptome dataset for target genes regulated by ctsTFs and performed GOBP/KEGG enrichment analysis on both ctsTFs and their downstream target genes. The results indicated that ctsTFs and their downstream target genes were consistently involved in cell-type–specific functions, such as cardiac muscle development in CMs, TGF-beta signaling in CFs, cell migration in ECs, and Toll-like receptor signaling in IMs (Fig. 4, *A* and *B* and Supplemental Table S3).

The expression patterns of ctsTFs and their target genes were strongly correlated. Specific TF-TG network genes were identified in pathways such as cardiac muscle cell differentiation in CMs (Pax3, Prox1, Rarg), TGF-beta signaling in CFs (Smad family, Tfdp1), endothelial cell migration in ECs (Sox18, Gata2, Meox2, Zfpm1), and myeloid leukocyte differentiation in IMs (Runx1, Rbpj, Irf4, Mef2c, Fos, Ikzf1) (Fig. 4, *C*–*F*). This high consistency between ctsTFs and their downstream target genes indicates the critical roles of ctsTFs in maintaining the key functions of each cell-type.

### Hierarchical Crosstalk Networks Among Cell-Types from Ligand–Receptor to TF–TG Interactions

We constructed a computational model to investigate the crosstalk among the four cell-types that are essential for maintaining CM identity. Using the CCCEXPLOR algorithms (46, 47), we analyzed signal transduction and protein interactions involving ligands, receptors, TFs, and TGs. We focused on ligands expressed predominantly in non-CM cells (NCMs), such as CFs, ECs, and IMs, along with their corresponding receptors, which were highly expressed in CMs. These interactions represented key elements of the cell-type crosstalk. CM-specific TFs and their downstream TGs were pivotal in maintaining CM identity. We combined significantly enriched signaling pathways using hypergeometric test (*p* < 0.05) and used Mann-Whitney U test to identify the shortest path lengths for crosstalk pathways regulating specific TFs. Interestingly, we found that the average shortest path length of the derived crosstalk signaling pathways regulating specific TFs was significantly shorter than those regulating all TFs (Fig. 4*G*), suggesting that NCMs maintain CM identity through specific ligand-receptor-ctsTF-TG signaling processes (Fig. 4*G*, Supplemental Table S3). This network revealed a division of labor among the four cell-types and highlighted the role of CM-NCMs crosstalk in maintaining cardiac function.

To further explore the role of hierarchical crosstalk networks in maintaining CM protein expression patterns, we cultured primary CMs and monitored their behavior over 0, 1, 3, 5, 7, and 9 days. During *in vitro* culturing, the morphology of CMs changed dramatically (Fig. 4*H*). We screened both TF patterns by catTFRE approach and proteome by Fast-seq approach (29, 45, 48), identifying 5840 proteins and 107 TFs in cultured CMs (Supplemental Fig. S4*A*). As the catTFRE can monitor the DNA-binding activity of the TFs quantitatively, the decrease in the identified TFs indicated inactivation of transcription in cultured CMs. Bioinformatics analysis showed that in freshly isolated CMs, TFs were primarily involved in cardiac-related pathways, but by day 3, their functions shifted toward cell cycle regulation (Supplemental Fig. S4*B*). Proteomic profiling reflected a similar shift, with oxidative phosphorylation and circulatory system processes downregulated over time, while cell cycle and apoptotic processes were upregulated (Fig. 4*I*). These findings suggest that *in vitro* culturing of primary CMs leads to a rapid loss of CMs identity in the absence of nonmyocytes, highlighting the importance of CM-NCM crosstalk in maintaining CM identity through TF activation.

### Region-Resolved Proteome of the Mouse Heart

**Fig. 5:**
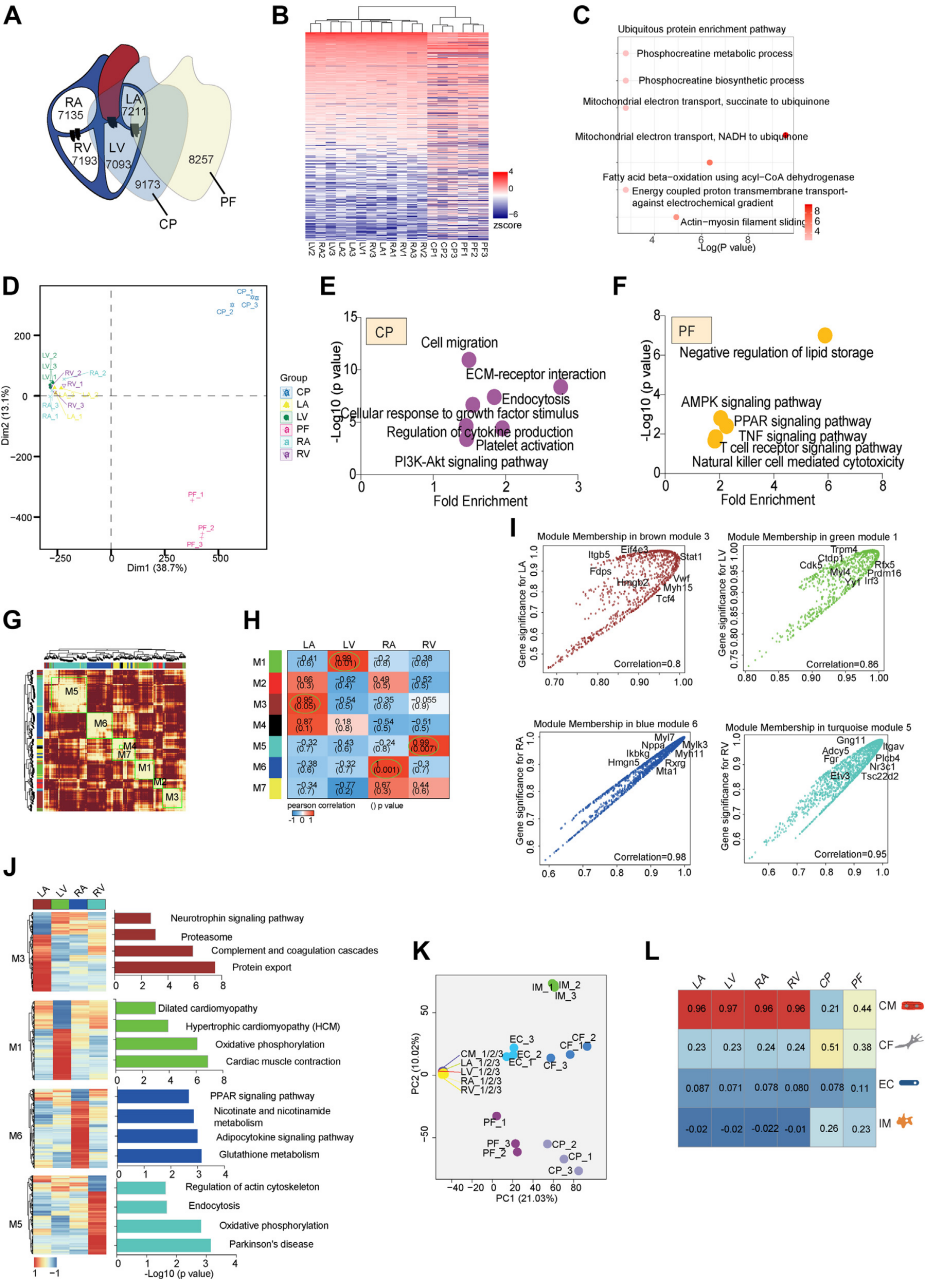
**Region-resolved mouse heart proteome.***A*, schematic illustration of region-resolved heart proteome, with the number of proteins detected in six anatomic regions. *B* and *C*, the heatmap representing ubiquitous proteins in six regions of heart (*B*). Median FOT value of proteins was calculated among six heart regions, and protein which median log_10_FOT were larger than −2 were determined as ubiquitous proteins. Their enrichment terms of ubiquitous proteins were annotated on the bubble plots (*C*). The y-axis of the bubble plot means GOBP/KEGG pathway. *D*, principal component analysis (PCA) of the protein expression patterns of major six heart regions. *E* and *F*, the bubble plots showed the GO/KEGG items enriched by CP- (*E*) and PF- (*F*) enhanced proteins. GO and KEGG pathway enrichment analyses were performed using the Database for DAVID website. *G*, network construction using consensus WGCNA on region-resolved proteome dataset. The color bars indicate assignment of modules. *H*, the heatmap indicates the correlation between seven assigned modules with the four regions of heart. The color bar on the *left* indicates assignment of modules. *I*, the scatter plots represent the significance of proteins’ enrichment in an assigned module versus the significance of proteins’ enrichment in a specific region of heart. X-axis and y-axis both represent correlation value ranging from 0 to 1. *J*, the heatmaps indicate the module-enriched proteins’ expression patterns in the four regions of heart with the GOBP/KEGG pathways on the *right*. *K*, principal component analysis (PCA) of the protein expression patterns of six major heart regions and four cell-types. *L*, the cell compositions of the six regions of heart, calculated by GSVA. The color bar indicates cell-type enrichment score.

Our deep coverage of cell-type–resolved heart proteome revealed critical biological processes and intercellular crosstalk that maintain these features across different cell-types. However, the precise anatomical resolution of proteome atlas was not yet explored. To address this question, we isolated six major regions of the mouse heart, LA, RA, LV, RV, CP, and PF (Fig. 5*A*). We identified 7093 to 9173 proteins in these regions, with a total of 11,995 proteins detected, achieving comprehensive coverage of the heart’s regional proteome (Fig. 5*A*, Supplemental Fig. S6*A* and Supplemental Table S4). To identify proteins with conserved expression across heart regions, we calculated the median FOT values across the six heart regions and defined ubiquitous proteins as those with median log_10_FOT values greater than −2 (Fig. 5*B*). Bioinformatic analysis showed that these ubiquitous proteins were enriched in housekeeping processes such as actin-myosin filament sliding and mitochondrial electron transport (Fig. 5*C*).

To further investigate differential protein expression across heart regions, we performed PCA analysis, which revealed clear coclustering of the LA, RA, LV, and RV, while CP and PF clustered separately (Fig. 5*D*, Supplemental Fig. S6*B*). Consistent with this observation, distinct biological functions were enriched in CP and PF regions. CP was associated with ECM–receptor interaction and cellular response to growth factor stimulus, whereas PF was enriched in immune pathways, such as T cell receptor signaling, natural killer cell–mediated cytotoxicity, and TNF signaling (Fig. 5, *E* and *F*, Supplemental Table S4).

The proteins enriched in atrioventricular areas (LA, RA, LV, RV) of the heart, including actin, troponin, titin, myosin light chain, and myosin heavy chain family, exhibited highly consistent expression patterns across the four regions (Supplemental Fig. S5*A*). To investigate the difference between atrioventricular area, we performed WGCNA, which identified seven main modules enriched in specific regions (Fig. 5*G*, Supplemental Table S4). Specifically, module M1 was enriched in LV (average correlation = 0.99, *p* value = 0.01), M3 enriched in LA (average correlation = 0.95, *p* value = 0.05), M5 enriched in RV (average correlation = 0.99, *p* value = 0.007), and M6 enriched in RA (average correlation = 1, *p* value = 0.001) (Fig. 5, *H* and *I*). GO analysis revealed distinct functional pathways for these modules (Fig. 5*J*). M1, associated with dilated cardiomyopathy and cardiac muscle contraction, contained enhanced proteins like Myl4, Trpm4, Irf3 in LV. M3, enriched in LA, was associated with neurotrophin signaling pathway contain proteins, including Stat1, Hmgb2, and Tcf4. M5, enriched in RV, contained enhanced proteins including Gng11, Itgav, and Etv3 and associated with cytoskeleton regulation pathways. Finally, M6, enriched in RA, was associated with PPAR signaling and glutathione metabolism, with proteins like Myl7, Nppa, and Myh11 (Fig. 5, *I* and *J*, Supplemental Fig. S5, *B*–*E*, and Supplemental Table S4).

We further integrated the region- and cell-type–resolved heart proteomes to construct a proteome-based cell to cell network for various heart regions. PCA analysis indicated that only CMs coclustered with the LA, RA, LV, and RV regions, whereas CFs, ECs, and IMs did not (Fig. 5*K*, Supplemental Fig. S6*C*). Gene set variation analysis (49) revealed that CMs predominantly expressed proteins enriched in the LA, RA, LV, and RV regions (Fig. 5*L*). Moreover, GOBP/KEGG functional analysis confirmed that the biological functions of a region were consistent with the functions of the major cell-type in that region. For example, the major function of LA/RA/LV/RV regions, oxidation-reduction process, was also the primary function of CMs.

In addition to studying cell-type– and region-specific proteomes separately, we also constructed a network showing how different cell-types cooperate across specific heart regions. As shown in Supplemental Fig. S6*D*, to execute the oxidation-reduction in LA/RA/LV/RV, CMs-enhanced proteins enriched in oxidation-reduction interact with CFs-enhanced proteins enriched in AMPK signaling pathway, ECs-enhanced proteins participate in cAMP signaling pathway, and IMs-enhanced proteins enriched in fructose metabolism, demonstrating the close relationship among various cell-types to maintain the function of LA/RA/LV/RV (Supplemental Table S4).

### Region-Resolved Human Heart Proteome in Dilated Cardiomyopathy Patients

**Fig. 6:**
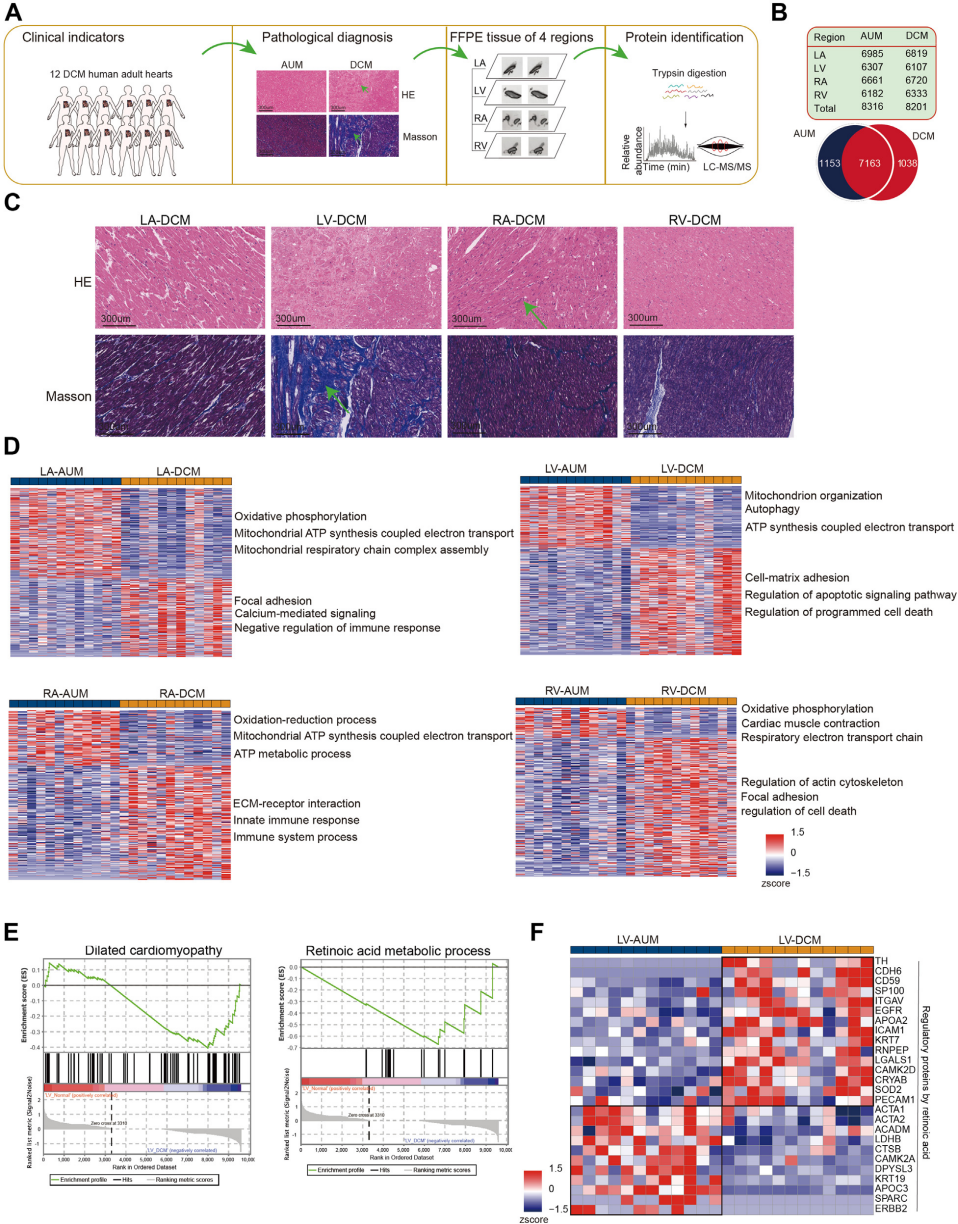
**The region-resolved human heart proteome in the dilated cardiomyopathy patients.***A*, schematic illustration of region-resolved human heart proteome in the dilated cardiomyopathy patients. *B*, Venn diagram and rectangle show the number of proteins detected in DCM area comparing to AUM area from human heart. *C*, microscopic pathology features of the four disease regions of human heart. HE staining illustrates apparent inflammation in atrial regions. Masson staining shows strong fibrosis in ventricular regions. Scale bars represent 300 μm. *D*, cluster heatmaps show expression patterns of proteins detected in the identical regions of human heart under DCM or AUM condition. The top GOBP/KEGG enriched by each cluster are shown on the *right*. The color bar indicates normalized z-scored iBAQ. *E*, GSEA enrichments showing dilated cardiomyopathy and retinoic acid metabolic process between AUM and DCM area. *F*, heatmap shows retinoic acid regulatory proteins in AUM and DCM area. The color bar indicates normalized z-scored iBAQ.

Our region-resolved mouse heart proteome revealed clear differences between heart regions as well as their connection to cell-type–resolved proteome. To explore whether heart disease shows similar proteome alterations across regions, we focused on DCM, a leading cause of heart failure, and conducted in-depth proteomic analysis of heart regions in DCM patients, comparing them with AUM tissue (Fig. 6*A*). The FFPE samples from 12 DCM patients were collected, and proteomes from LA, RA, LV, RV regions were analyzed. Proteome identification ranged from 6107 proteins in LV to 6819 proteins in LA, with a total of 8201 proteins identified in DCM area. In AUM area, proteome identification ranged from 6182 proteins in RV to 6985 proteins in LA, with a total of 8316 proteins identified (Fig. 6*B*). Pathological examination revealed that RA exhibited obvious inflammatory response, while LV showed extensive fibrosis (Fig. 6*C*). Consistent with the pathological observations, proteome data showed that, for DCM area, calcium-mediated signaling was upregulated in the LA, innate immune response was upregulated in the RA, LV contained apoptotic signaling, and RV featured focal adhesion (Fig. 6*D*, Supplemental Table S5). Since DCM particularly affects the LV (50, 51), we analyzed the differential expression of proteins between DCM area and AUM area in LV. Gene set enrichment analysis indicated that proteins enriched in DCM area were predominantly related to DCM and retinoic acid metabolic processes (Fig. 6*E*). We further validated regulatory targets of retinoic acid, previously reported in the literature (52), within our proteome data. It is notable that these retinoic acid targets were significantly changed in LV samples of DCM, including 15 upregulated proteins and 11 downregulated proteins (Fig. 6*F*, Supplemental Table S5). This phenomenon indicated retinoic acid signaling pathway may play a critical role in DCM.

### The Protective Role of Retinoic Acid in Heart Failure

**Fig. 7:**
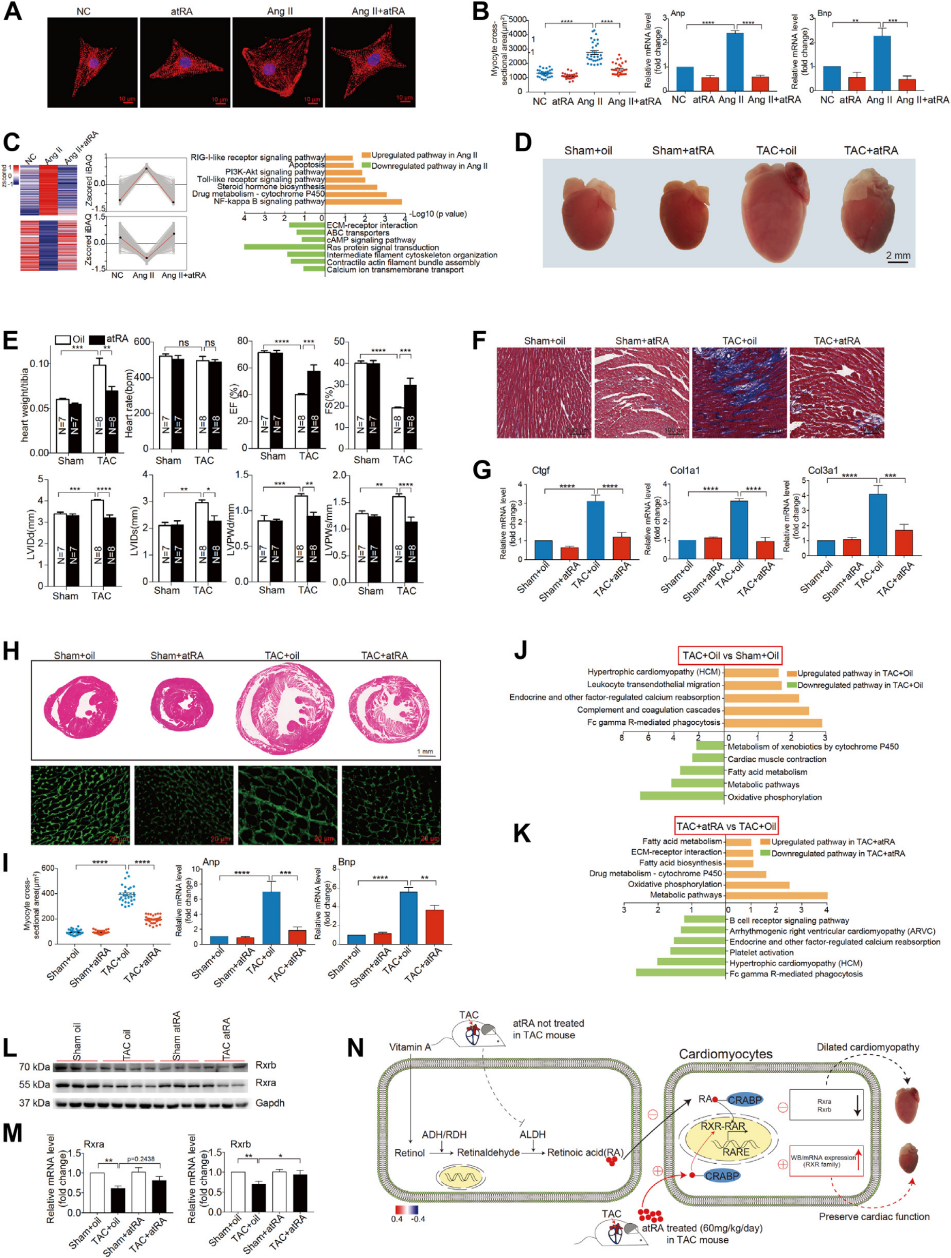
**The protective role of atRA in heart failure.***A*, α-Actinin staining was performed to identify NRCMs. Representative images and quantification of cell size of total 30 microscope fields in each group (group1: NRCMs, group2: NRCMs treated with atRA, group3: NRCM stimulated with Ang II, and group4: NRCM stimulated with Ang II then treated with atRA) are shown. *B*, the boxplot shows the myocyte cross-sectional area in the four groups. The mRNA levels of hypertrophic genes (*Anp, Bnp*) were determined using qRT-PCR. n = 4 biological replicates for each group. ∗*p* < 0.05, ∗∗*p* < 0.01, ∗∗∗*p* < 0.001, ∗∗∗∗*p* < 0.0001, ns: not significant. *C*, the cluster heatmaps indicate the expression patterns of two cluster of proteins detected in NRCM under different conditions (Ang II nonstimulate, Ang II stimulated, Ang II stimulated with atRA treatment). The color bar indicates normalized z-scored iBAQ. The top GOBP terms enriched by each cluster are shown on the *right*. *D*, representative global heart photographs of four mice are shown (scale bar represents 2 mm). *E*, the bar plot shows the heart weight/tibia, heart rate, LV ejection fraction, fractional shortening, LV internal dimension at end-diastole, LV internal dimension at end-systole, LV anterior wall thickness at end-diastole, LV posterior wall thickness at end-systole, all measurements are shown as mean ± SEM. n = 7 biological replicates in Sham group and n = 8 biological replicates in TAC group. *F*, Masson staining showed strong fibrosis in ventricular regions. Scale bars represent 100 μm. *G*, the mRNA levels of *Ctgf, Cola1*, *and Col3a1* were determined using qRT-PCR. n = 6 biologically replicates for each group. ∗*p* < 0.05, ∗∗*p* < 0.01, ∗∗∗*p* < 0.001, ∗∗∗∗*p* < 0.0001, ns: not significant. *H*, hematoxylin-eosin (H&E, scale bar = 1 mm) staining and wheat germ agglutinin (WGA, scale bar = 30 μm) staining were performed to determine the hypertrophic degree of the hearts. *I*, the boxplot shows the myocyte cross-sectional area in the four groups. The mRNA levels of hypertrophic genes (*Anp, Bnp*) were determined using qRT-PCR. n = 6 biologically replicates for each group. ∗*p* < 0.05, ∗∗*p* < 0.01, ∗∗∗*p* < 0.001, ∗∗∗∗*p* < 0.0001, ns: not significant. *J*, the bar plot indicates the GOBP enriched in the proteins upregulated in TAC group (*orange*) and downregulated in TAC group (*green*), compared with sham oil group. *K*, the bar plot shows the GOBP enriched by the proteins upregulated in TAC mice treated with atRA (*orange*) and downregulated in TAC mice treated with atRA (*green*), compared with TAC oil group. *L*, the western blots show the expression patterns of Rxra, Rxrb, and Gapdh in the four groups (group1: WT mice treated with corn oil, group2: TAC mice treated with corn oil, group3: WT mice treated with atRA, Group4: TAC mice treated with atRA). *M*, The mRNA level of *Rxra and Rxrb* were determined using qRT-PCR. n = 7 biologically replicates for each group. ∗*p* < 0.05, ∗∗*p* < 0.01, ∗∗∗*p* < 0.001, ∗∗∗∗*p* < 0.0001, ns: not significant. *N*, the hypothetical mechanism of atRA rescuing heart from cardiac hypertrophy. Under physiological conditions, retinol is oxidized to retinaldehyde by either alcohol dehydrogenase or retinol dehydrogenase (RDH), and retinaldehyde is oxidized to RA by aldehyde dehydrogenase (ALDH). RA is then released and taken up by cardiomyocytes and cellular-RA–binding protein (CRABP) facilitates RA to transport to the nucleus where RA can bind the RA receptor (RAR). When mouse is treated with atRA (60 mg/kg/day) for 4 weeks after TAC surgery, cardiac hypertrophy is relieved. The atRA may act through the activation of Rxr family and maintains cardiac function.

To examine the functional role of retinoic acid signaling pathway in DCM, we constructed an AngII-induced cardiac hypertrophy model using primary cultured NRCMs. Cells were treated with or without 1 μM atRA (Supplemental Fig. S7*A*). As shown in Figure 7, *A* and *B*, and Supplemental Fig. S7*B*, atRA treatment significantly reduced cardiomyocyte size and downregulated the expression of hypertrophic genes (*Anp, Bnp, Saa*, *and β-Mhc*), indicating that atRA effectively abolished AngII-induced hypertrophy in NRCMs. To obtain a comprehensive perspective of the changes induced by atRA, we performed mass spectrometry to profile the proteomes of AngII-stimulated NRCMs treated with or without atRA. In total, 10,164 proteins were identified, with 1666 proteins downregulated in AngII-stimulated NRCMs compared to nonstimulated NRCMs and 1293 proteins upregulated in atRA-treated AngII-stimulated NRCMs compared to untreated AngII-stimulated NRCMs (Supplemental Fig. S7*C*). GO analysis revealed that proteins involved in calcium ion transmembrane transport and contractile actin filament bundle assembly were significantly downregulated after AngII stimulation and upregulated by atRA treatment. Additionally, proteins enriched in drug metabolism, cytochrome P450 and apoptosis, which were elevated after AngII stimulation, were reduced after atRA treatment (Fig. 7*C*, Supplemental Table S7). These findings suggest that atRA may inhibit apoptosis and functional impairment of NRCMs caused by AngII stimulation, indicating its potential protective role against cardiac hypertrophy.

To further evaluate whether atRA exerts an antihypertrophic effect *in vivo*, we constructed a TAC-induced cardiac hypertrophy mouse model. Mice were treated with atRA (60 mg/kg/day) for four weeks following TAC surgery, and corn oil–treated TAC mice served as controls. After four weeks, control mice exhibited significant increases in key hypertrophic parameters, while atRA treatment effectively reduced these parameters and improved cardiac function, as evidenced by a lower heart weight-to-tibia ratio and maintained ejection fraction (Fig. 7, *D* and *E*, Supplemental Fig. S7*D*, and Supplemental Table S7). Masson’s trichrome staining revealed that atRA treatment significantly reduced fibrosis in the hearts of TAC mice compared to controls (Fig. 7*F*). These protective effects were further confirmed by H&E staining and WGA staining, as well as reduced mRNA expression of key hypertrophy genes (Fig. 7, *H* and *I*). Hearts from atRA-treated group showed no significant pathological feature, whereas control group hearts exhibited noticeable pathological hypertrophy. Additionally, fibrotic genes (*Ctgf*, *Col1a1*, *Col3a1*) and hypertrophic genes (*Anp, Bnp*, *Mhy7, Rcana 1.4*, *Acta1*) were downregulated after atRA treatment (Fig. 7, *G* and *I*, and Supplemental Fig. S7*E*). Based on these findings, we conducted proteomic analysis on three groups of mice (group1: sham with oil treatment, group2: TAC with oil treatment, group3: TAC with atRA treatment). We quantified 4659 proteins in sham group, 5556 proteins in TAC group, 4962 proteins in TAC with atRA treatment group (Supplementary Fig. S7, *F* and *G*). We then compared protein expression patterns across the three groups and selected differentially expressed proteins for further analysis. We found that protein upregulated in TAC group were enriched in leukocyte transendothelial migration and hypertrophic cardiomyopathy pathways, while downregulated proteins participated in cardiac muscle contraction and oxidative phosphorylation, indicating significant loss of heart function after TAC (Fig. 7*J*, and Supplemental Fig. S7*H*). Conversely, in the atRA-treated TAC group, proteins associated with oxidative phosphorylation were upregulated, and proteins involved in platelet activation and hypertrophic cardiomyopathy were downregulated, indicating that atRA rescued heart function by preserving fundamental cardiac processes and metabolic pathways (Fig. 7*K* and Supplemental Fig. S7*I*). Notably, atRA receptor Rxrb was upregulated at both protein level and mRNA levels in the atRA-treated group compared to control group, while Rxra showed no significant change (Fig. 7, *L* and *M*). As Rxrb has been reported to play a vital role in regulating heart function (53, 54, 55), we hypothesize that the cardiac protective effects of atRA are mediated, at least in part, by the activation of Rxr family, maintaining cardiac function (Fig. 7*N*).

## Discussion

The progress of MS-based proteomics has provided unprecedented accuracy in quantifying protein levels, offering novel insights into biological processes. Approximately 15% to 70% of the variation in protein levels can be attributed to posttranscriptional and posttranslation regulation, as well as measurement errors (56). Thus, the regulation of the protein expression level may play an even more dominant role than transcriptional regulation in certain physiological responses.

While organs are composed of different cell-types organized in precise spatiotemporal arrangements, current organ proteome datasets often reflect mixed cell-types and regions, limiting the resolution needed to fully understand cellular functions. To address this, the field has increasingly focused on cell-type– and region-resolved proteomics, which have become a critical area of life sciences research. In this study, we present a high-resolution, reproducible cell-type–, and region- resolved heart proteome and identify 11,794 proteins of cell-type–resolved and 11,995 proteins of region-resolved, marking it the deepest proteomic exploration of the heart to date.

Building upon our previous research with human cardiovascular progenitor cells and human cardiomyocytes in coculture models, we observed substantial plasticity and paracrine diversity between these cell-types (57). Similarly, in primary isolated adult mouse hearts, we found that CMs performed major functions, including muscle contraction and ATP metabolism, while nonmyocytes (CFs, ECs, and IMs) regulated CM’s function through signal transduction. For instance, ECs secrete nitric oxide, which transmitted signals to CMs via the nitric oxide–cyclic GMP pathway, playing a well-documented role in modulating CM function, particularly through acute effects on muscle contraction. Our findings highlight a hierarchical crosstalk network that underlies the functional division between different cell-types, ensuring efficient cardiac performance. TFs are pivotal regulators of biological processes. The deep proteome coverage in this study enabled the detection of 479 TFs in mouse heart. By integrating TF patterns with proteome profiling, we constructed TF-TG networks that were closely linked to cell-type–specific functions and cellular identities.

In addition to cell-type specificity, the region-resolved heart proteome provided another layer of understanding. The central regions of the heart (LA, RA, LV, and RV) were associated with core cardiac functions, while peripheral regions, such as the CP and the PF, played regulatory roles. The CP was enriched with ECM–receptor interaction, steroid hormone biosynthesis, while the PF was involved in immune pathways. This regional specialization suggests that different cell-types dominate the functions of distinct regions, organized in precise spatial patterns.

We extended our analysis to diseased hearts, focusing on 12 DCM FFPE samples. Despite the challenges associated with FFPE sample degradation, previous studies have demonstrated the feasibility of proteomic analysis in these samples (58, 59, 60, 61). We identified 9594 proteins at region-resolved level in DCM samples which revealed that downregulated proteins were mainly involved in calcium ion transport and cardiac muscle contraction, suggesting a loss of essential cardiac functions in DCM. Due to the limited availability of normal human heart tissue, we used AUM area as control. With the lack of conventional controls, we employed H&E staining and Masson's trichrome staining which can reveal several characteristic features in DCM (62). Importantly, the cardiomyocytes in the AUM areas were confirmed to be completely normal, and regions without fibrosis were designated as AUM areas, thereby providing a reasonable comparative reference in the absence of healthy controls.

Ni *et al*. previously re-analyzed public proteomic data from six human DCM samples (63, 64), proposing that low cardiac ATRA attenuates the expression of critical ATRA-dependent gene programs in heart failure. In our study, we constructed a region-resolved heart proteome from 12 Chinese DCM samples, identifying 9594 proteins. This significantly surpasses the 3410 proteins identified by Ni *et al* (Supplemental Fig. S6*E*). Moreover, based on 532 known regulatory targets of retinoic acid (52), we identified 218 proteins, compared to 81 proteins reported by Ni *et al* (Supplemental Fig. S6*F*).

While cross-species comparisons between mouse and human are not perfect, previous studies have shown significant biological conservation between these species (65, 66). To evaluate if the heart proteome of mouse could be used as a reference database for human, we compared our data with published region-resolved human heart proteome (25). Over 70% of human heart proteins were detected in our dataset, and the GOBP/KEGG analysis of the four region-enhanced genes indicated that dominant functions of four regions were conserved in both human and mouse (Supplemental Fig. S8*A*). These support the utility of mouse heart proteomes as reference datasets for human DCM study.

Retinoic acid, an active derivative of vitamin A, plays a crucial role in cardiovascular function (67, 68). Previous studies have demonstrated RA’s protective effects against apoptosis induced by Ang II and mechanical stretch, though evidence for its role in rescuing cardiac hypertrophy *in vivo* has been limited (69). Our findings contribute to the growing evidence supporting the therapeutic potential of RXR activation in heart failure, while recognizing previous studies that have explored its role in cardiac hypertrophy (69). Proteomic analysis further illustrated that atRA rescued heart failure through enhancing the expression of proteins involved in muscle contraction while reducing the expression of proteins-associated inflammation.

Overall, our study presents a detailed cell-type– and region-resolved mouse heart proteome, providing in-depth coverage comparable to RNA-seq. This dataset uncovers the division of labor among major heart cell-types, highlighting the critical role of TF-TG networks in maintaining heart function. The mapping of the region-resolved and cell-type–resolved proteome allowed us to achieve a better understanding of the precise spatial order of different cell-types functioned in physiology and pathology conditions. Additionally, we demonstrated the protective effects of atRA in heart failure, opening new avenues for therapeutic intervention. This comprehensive heart proteome map serves as a valuable resource for future cardiac research.

## Data Availability

The MS proteomics raw data have been deposited to the ProteomeXchange Consortium (http://proteomecentral.proteomexchange.org) *via* the iProX partner repository with the dataset identifier PXD060863 under Project ID IPX0002590000. The transcriptomics data is accessible in NODE (https://www.biosino.org/node) under the accession number OEP00005637.

## Supporting information

This article contains supporting Information.

## Conflict of Interest

The authors declare no competing interests.
