# Supplementary figures and images for: Region- and Cell-type–Resolved Multiomic Atlas of the Heart
Source: Mol Cell Proteomics. 2025 Feb 5;24(5):100922. doi: 10.1016/j.mcpro.2025.100922 (PMC12139502; doi:10.1016/j.mcpro.2025.100922)

Supplementary Figure 1

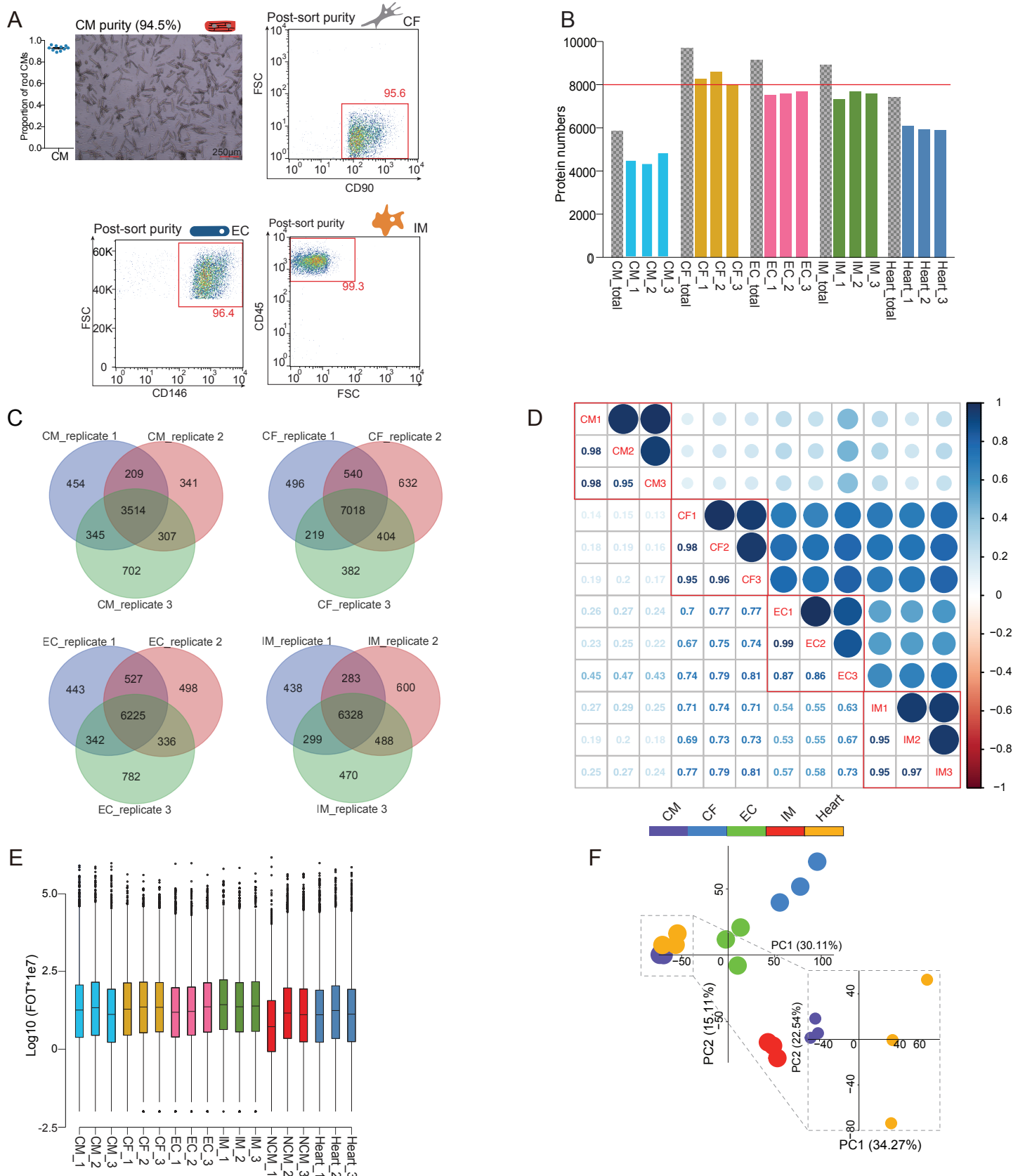

Supplement: Supplementary Figure 1 [file mmc8.pdf]

# Supplementary Figure 2

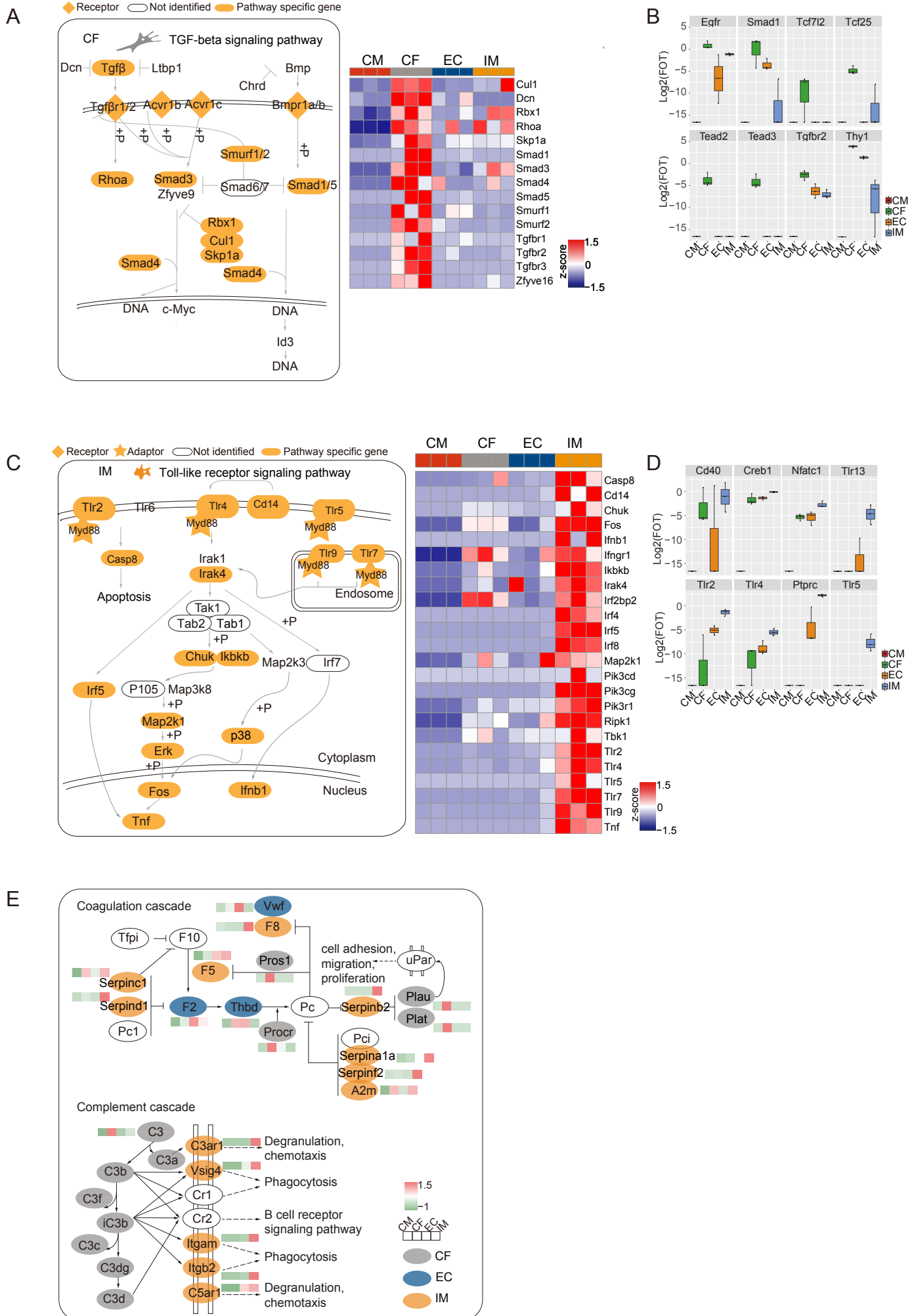

Supplement: Supplementary Figure 2 [file mmc9.pdf]

Supplementary Figure 3

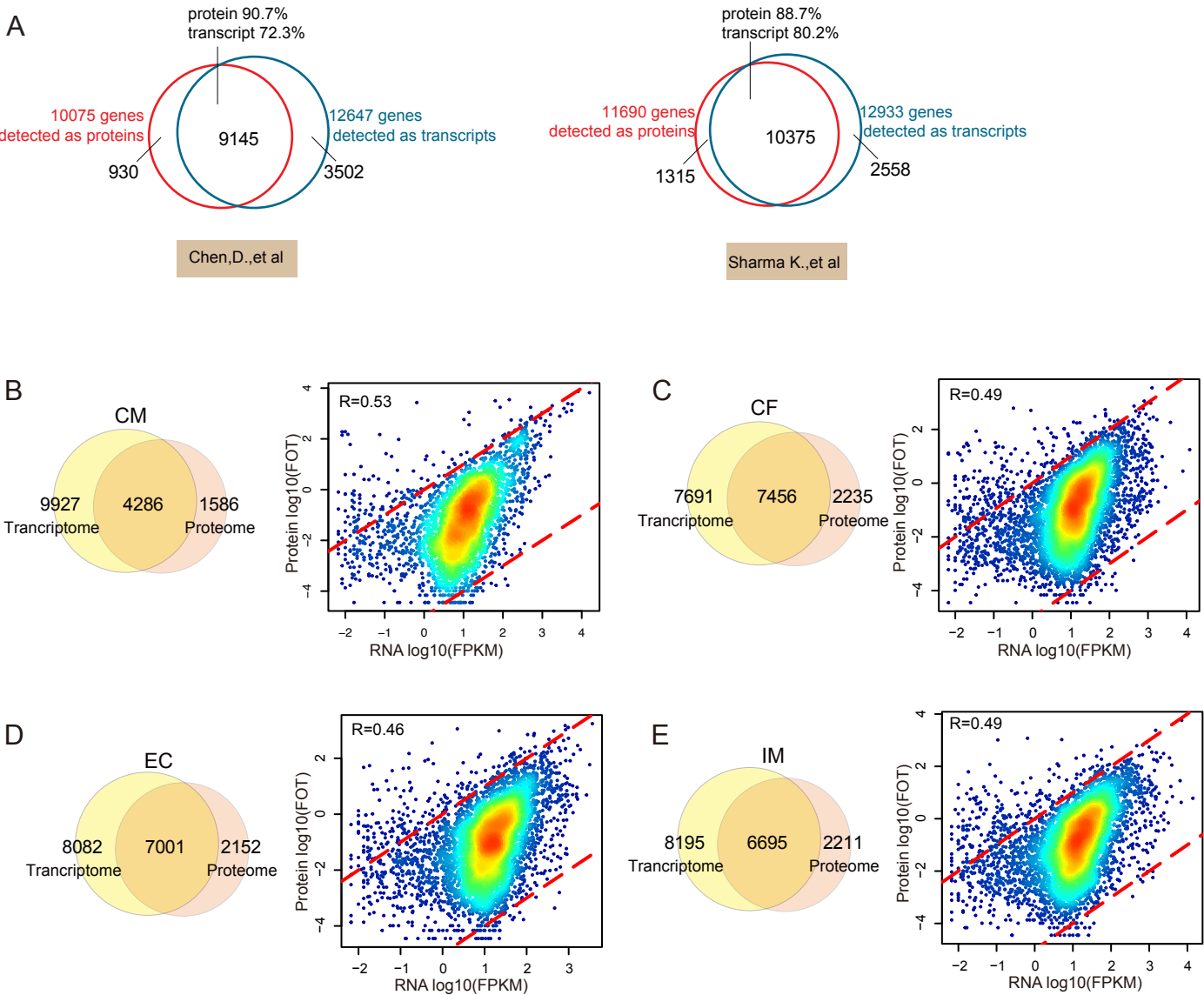

Supplement: Supplementary Figure 3 [file mmc10.pdf]

Supplementary Figure 4

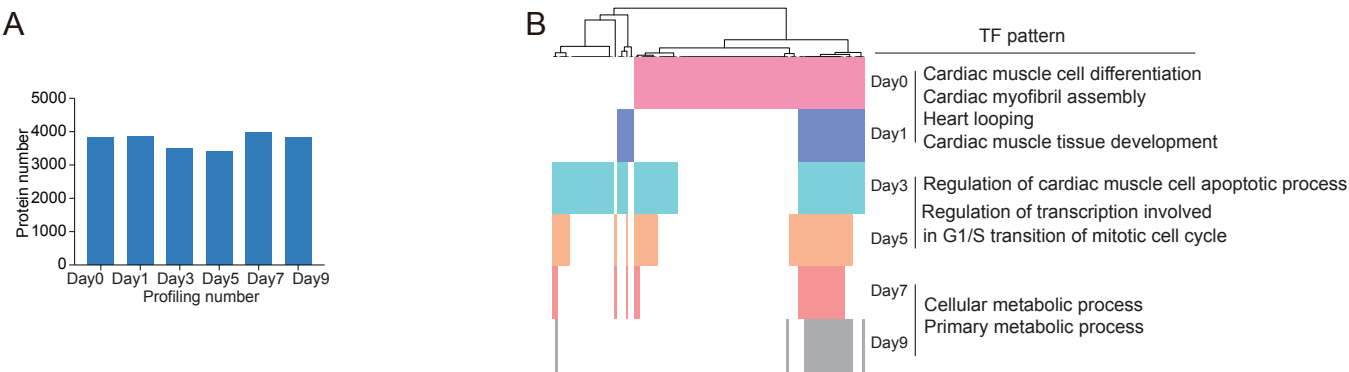

Supplement: Supplementary Figure 4 [file mmc11.pdf]

Supplementary Figure 5

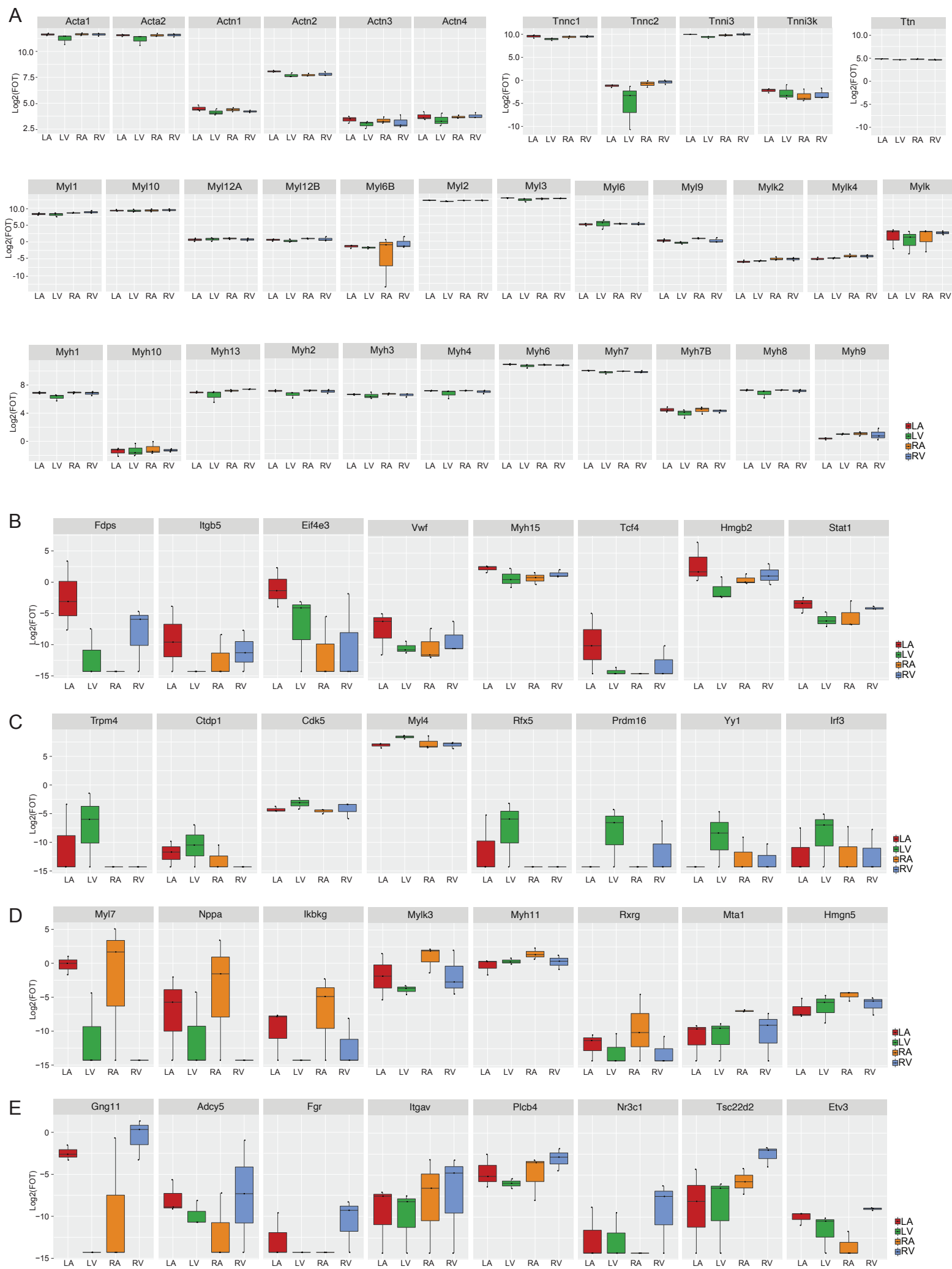

Supplement: Supplementary Figure 5 [file mmc12.pdf]

Supplementary Figure 6

A

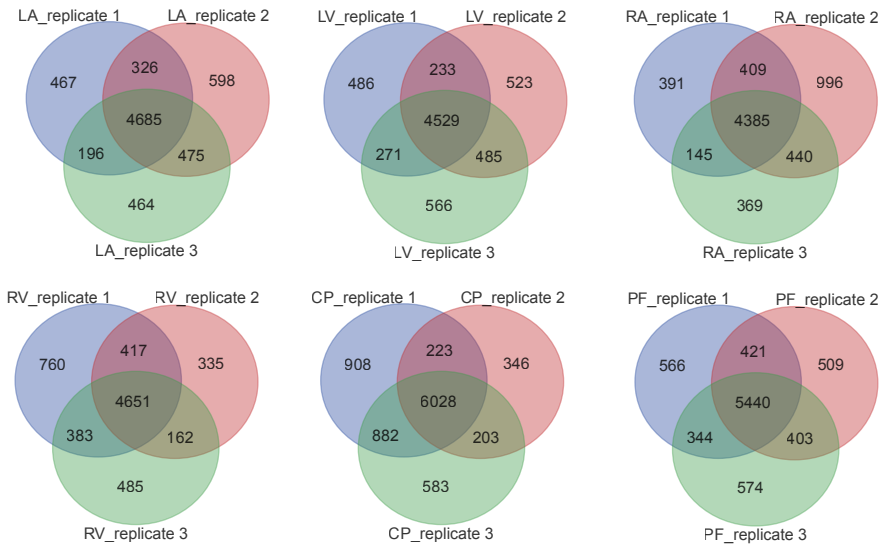

B

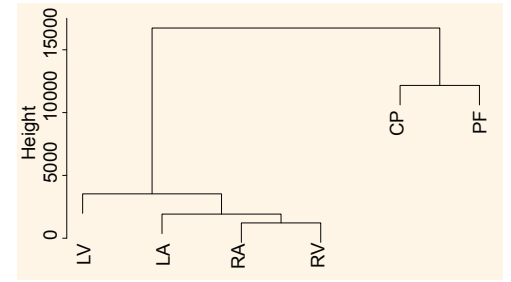

C

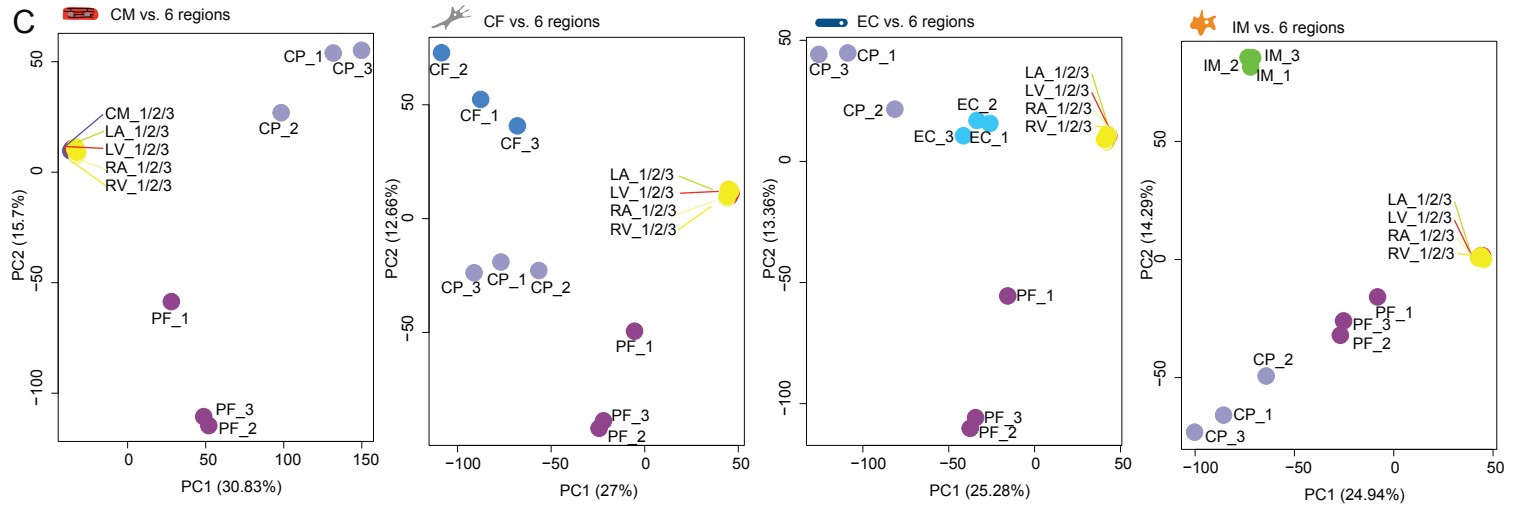

D

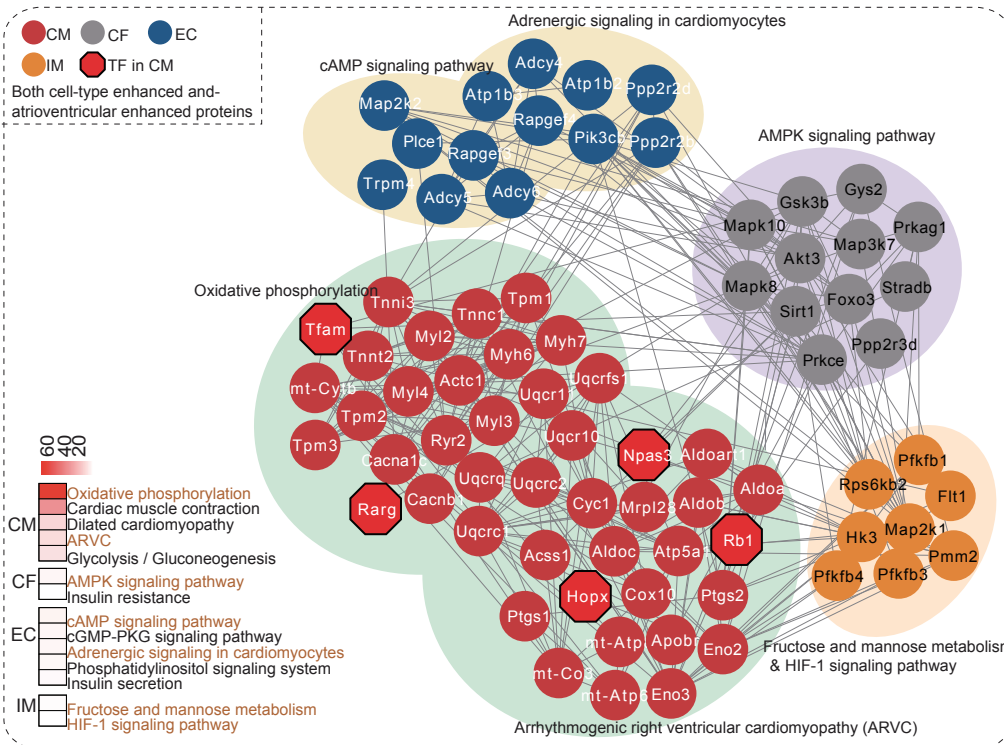

E

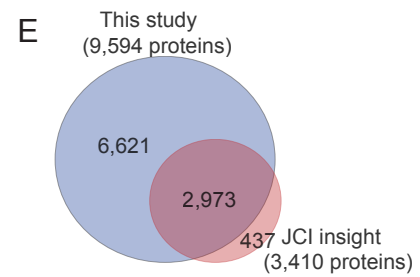

F atRA targets in this study (218 proteins)

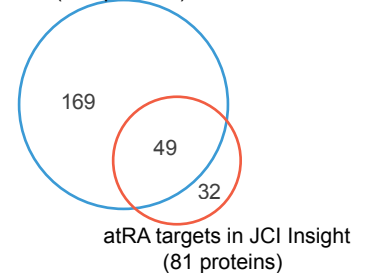

Supplement: Supplementary Figure 6 [file mmc13.pdf]

Supplementary Figure 7

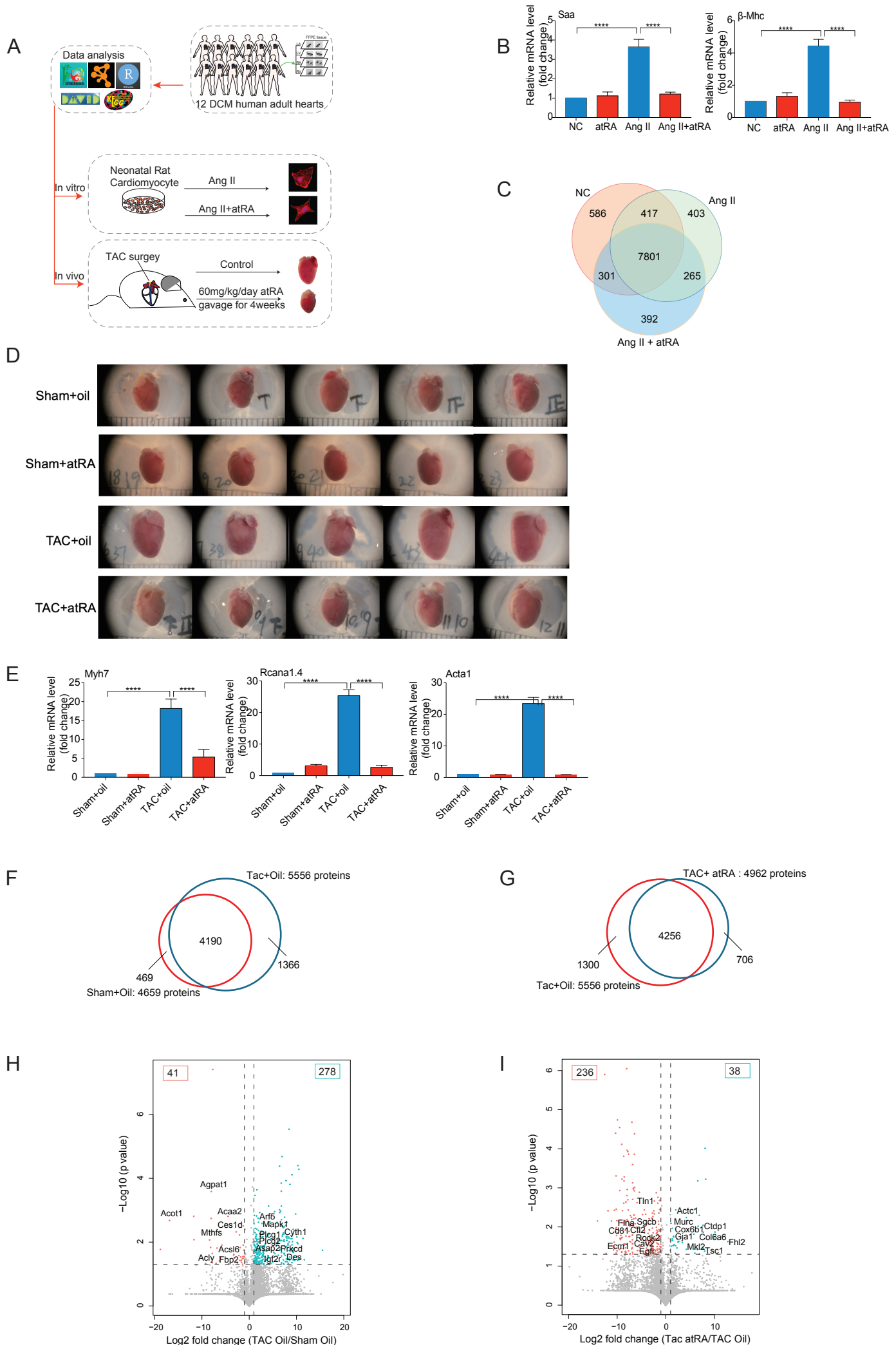

Supplement: Supplementary Figure 7 [file mmc14.pdf]

Supplementary Figure 8

A

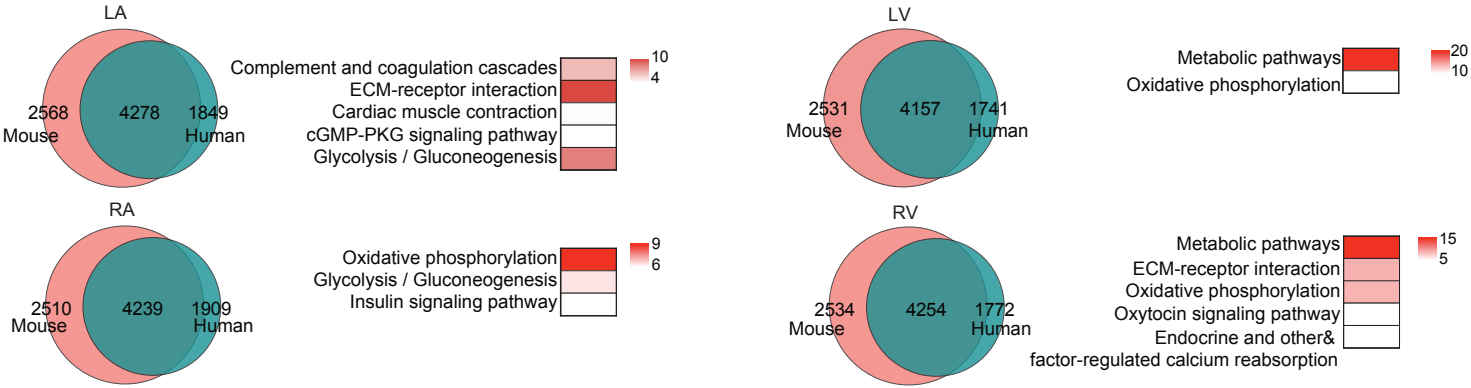

Supplement: Supplementary Figure 8 [file mmc15.pdf]
