# Supplementary material for: Region- and Cell-type–Resolved Multiomic Atlas of the Heart
Source: Mol Cell Proteomics. 2025 Feb 5;24(5):100922. doi: 10.1016/j.mcpro.2025.100922 (PMC12139502; doi:10.1016/j.mcpro.2025.100922)
Supplement: Supplementary Figure Legends [file mmc16.docx]

**Supplementary Figure Legends**

**Supplementary Figure 1. Proteome of the major heart cell-types, related to Figure 1.**

1. Representative image showing the high purity of cardiac myocytes isolated from mouse heart (94.5%). Non-myocytes were stained with known cell markers: CD90 (cardiac fibroblast), CD146 (endothelial cell) and CD45 (immune cell). Scatter plots show the post-sort purities of CFs, ECs and IMs were 95.6%, 96.4% and 99.3%, respectively.
2. The bar plot indicates the number of Gene Products (GPs) detected in cell-type resolved heart proteome.
3. Venn diagram of the identified protein numbers among four cell-types in mice.
4. The matrix of correlation plots revealing very high correlation between proteins' intensities in triplicates (Pearson correlation coefficient 0.80-0.97 between cell-types).
5. Boxplot illustrating the protein abundance of 18 experiments.
6. Principal component analysis (PCA) of the protein expression patterns of major heart cell-types.

# **Supplementary Figure 2. Proteome of the four major heart cell-types, related to Figure 2.**

A-D. The protein expression patterns of TGF-beta signaling pathway, Toll-like receptor signaling pathway in the proteome of CFs and IMs. The heatmaps represent the expression patterns of critical proteins participate in the pathway. The color bar of heatmap indicates normalized z-scored iBAQ. The boxplots show cell-type enhanced proteins in CFs (B) and IMs (D), including transcription factors and proteins functioned in cell-type.

E.Coagulation and complement pathway in the proteome of the four major heart cell-types. The color bar indicates normalized z-scored iBAQ.

**Supplementary Figure 3. Transcriptome of the four major heart cell-types, related to Figure 3.**

A. The Venn Plots show the comparison of coverage of proteome and transcriptome in previous studies (Chen D., et al, PMID: 27562671, Sharma K., et al, PMID: 26523646).

B-E. Venn diagram of the identified gene numbers at the protein and mRNA level for the indicated cell-types. The thermodynamic chart shows correlation between protein and mRNA levels for the major four cell-types. R is the Spearman correlation coefficient.

# **Supplementary Figure 4. TF sub-proteome and proteome of in vitro cultured cardiac myocytes, related to Figure 4.**

A. Bar chart showing protein numbers of TF sub-proteome level for six time points.

B. The cluster heatmap showing the DNA-binding activity patterns of the three clusters of transcription factors identified in the TF sub-proteome of in vitro cultured cardiac myocytes, with GO terms enriched in each cluster of proteins on the right.

# **Supplementary Figure 5. Region resolved heart proteome, related to Figure 5.**

# The boxplots showing the expression patterns of ubiquitous proteins in the four regions of heart (LV, LA, RA and RV).

B-E. The boxplots showing the expression patterns of proteins which show enhanced expression in the four regions of heart (LA, LV, RA and RV).

# **Supplementary Figure 6. Cell-type function division in the various heart regions of healthy mice (related to Figure 5) and proteome for DCM patients (related to Figure 6).**

1. Venn diagram of the identified protein numbers among six regions in mice.
2. The hierarchical clustering of the samples from 6 regions of heart, reveals two distinct clusters representing cluster 1: LV, LA, RV and RA, cluster 2: CP and PF.
3. The scatter plots from *left* to *right* show the principal component analysis (PCA) of the 6 major heart regions with CM (*left*), with CF (*middle-left*), and with EC (*middle-right*), and with IM (*right*) based on their proteomic expression profiles.
4. The string network showing the proteins interaction among cell-type enhanced protein expressed mainly in atrium and ventricle. The heatmap below shows the GOBP/KEGG processes enriched in cell-type enhanced proteins expressed mainly in atrium and ventricle.
5. The Venn diagram shows the comparison of coverage of proteome in our study and previous reported study (**JCI Insight. 2021 Apr 22; 6(8): e137593**).
6. The Venn diagram shows the comparison of retinoic acid targets in our study and previous reported study (**JCI Insight. 2021 Apr 22; 6(8): e137593**).

**Supplementary Figure 7. Functional studies of atRA, related to Figure 7.**

1. The experimental design of functional study of atRA by *in vivo* and *in vitro* models.
2. The mRNA levels of *Saa, β-Mhc* were determined using qRT-PCR. n = 4 biologically replicates for each group. *P<0.05, **P<0.01, ***P<0.001.
3. The Venn plot shows the proteins detected in NRCM under different conditions (Ang II non-stimulate, Ang II stimulated and atRA treated Ang II stimulated).
4. All heart photographs of four groups are shown. Sham+oli: wild type mice treated with corn oil, Sham+atRA: wild type mice treated with 60mg/kg atRA, TAC+oil: TAC mice treated with corn oil, TAC+atRA: TAC mice treated with 60mg/kg atRA.
5. The mRNA level of *Myh7, Rcana 1.4 and Acta1* were determined using qRT-PCR. n = 6 biologically replicates for each group. *P<0.05, **P<0.01, ***P<0.001.
6. The Venn plot shows the proteins detected in heart samples from mice under different conditions (sham group treated with corn oil, TAC group treated with corn oil).
7. The Venn plot shows the proteins detected in heart samples from mice under different conditions (TAC group treated with corn oil, TAC group treated with atRA).
8. The volcano plot shows the proteins detected in heart samples from mice under different conditions (sham group treated with corn oil, TAC group treated with corn oil).
9. The volcano plot shows the proteins detected in heart samples from mice under different conditions (TAC group treated with corn oil, TAC group treated with atRA).

# **Supplementary Figure 8. Comparison between region-resolved proteome of human and mouse heart.**

1. The Venn diagram of the protein numbers identified in mouse (this paper) and in human (Doll et, al.’s work, PMID: 29133944) for an identical region of heart. The heatmap reveals the consistence of GO/pathway of an identical region between human or mouse heart. The color bar indicated normalized p-value.
